# m6Am Methyltransferase PCIF1 Promotes LPP3 Mediated Phosphatidic Acid Metabolism and Renal Cell Carcinoma Progression

**DOI:** 10.1002/advs.202404033

**Published:** 2024-10-18

**Authors:** Wenqin Luo, Zhehao Xu, Fan Li, Lifeng Ding, Ruyue Wang, Yudong Lin, Xudong Mao, Xianjiong Chen, Yang Li, Zeyi Lu, Haiyun Xie, Huan Wang, Ziwei Zhu, Yi Lu, Luying Guo, Xiaojing Yu, Liqun Xia, Housheng Hansen He, Gonghui Li

**Affiliations:** ^1^ Department of Urology Sir Run Run Shaw Hospital Zhejiang University School of Medicine Hangzhou 310016 China; ^2^ Kidney Disease Center of First Affiliated Hospital Zhejiang University School of Medicine Hangzhou 310000 China; ^3^ Department of Radiology Sir Run Run Shaw hospital Zhejiang University School of Medicine Hangzhou 310016 China; ^4^ Princess Margaret Cancer Centre University Health Network Toronto Ontario M5G 1L7 Canada; ^5^ Department of Medical Biophysics University of Toronto Toronto Ontario M5G 1L7 Canada

**Keywords:** mitochondrial dynamics, N6,2′‐O‐dimethyladenosine, PCIF1, phosphatidic acid metabolism, renal cell carcinoma

## Abstract

N6‐methyl‐2′‐O‐methyladenosine (m6Am), occurring adjacent to the 7‐methylguanosine (m7G) cap structure and catalyzed by the newly identified writer PCIF1 (phosphorylated CTD interacting factor 1), has been implicated in the pathogenesis of various diseases. However, its involvement in renal cell carcinoma (RCC) remains unexplored. Here, significant upregulation of PCIF1 and m6Am levels in RCC tissues are identified, unveiling their oncogenic roles both in vitro and in vivo. Mechanically, employing m6Am‐Exo‐Seq, LPP3 (phospholipid phosphatase 3) mRNA is identified as a key downstream target whose translation is enhanced by m6Am modification. Furthermore, LPP3 is revealed as a key regulator of phosphatidic acid metabolism, critical for preventing its accumulation in mitochondria and facilitating mitochondrial fission. Consequently, Inhibition of the PCIF1/LPP3 axis significantly altered mitochondrial morphology and reduced RCC tumor progression. In addition, depletion of PCIF1 sensitizes RCC to sunitinib treatment. This study highlights the intricate interplay between m6Am modification, phosphatidic acid metabolism, and mitochondrial dynamics, offering a promising therapeutic avenue for RCC.

## Graphical abstract

m6Am, an RNA modification adjacent to the 7‐methylguanosine cap structure, has been implicated in the development of various diseases. This study explores the oncogenic role of m6Am and its ‘writer’ enzyme, PCIF1, in renal cell carcinoma. It further identifies a novel connection between m6Am, phosphatidic acid metabolism, and mitochondrial dynamics in the context of cancer.

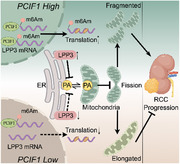

## 1 Introduction

Chemical modifications on RNA have garnered significant attention in the field of epigenomics due to their pivotal role in various RNA biological processes. Among the over 160 known RNA modifications across multiple species,^[^ ^1^ ^]^ N^6^‐methyladenosine (m^6^A) stands out as one of the most extensively studied, prevalent within mRNA adenosines and notably enriched on 3’ untranslated region (3’UTR) of mature mRNA.^[^ ^2^ ^]^ The reversible and dynamic nature underscores its significant regulatory role in both physiological and pathological contexts.^[^ ^3^, ^4^, ^5^ ^]^ In addition to m6A, another analogous modification, N6,2′‐O‐dimethyladenosine (m6Am), positioned exclusively at the mRNA transcription start nucleotide adjacent to 7‐methylguanosine (m7G) cap structure, has recently gained attention.^[^ ^6^, ^7^ ^]^ Despite its discovery several decades ago, research into m6Am has only recently intensified. Notably, He et al. revealed fat mass and obesity‐associated protein (FTO)'s dual role as a demethylase of both m6A and m6Am,^[^ ^8^ ^]^ while Suzuki et al.T identified PCIF1 as the writer of this modification.^[^ ^9^ ^]^ Functional studies have unveiled diverse roles of m6Am under different biological contexts.^[^ ^9^, ^10^, ^11^ ^]^ Emerging evidence also suggests its involvement in disease progression, including cancer, obesity, and viral infection,^[^ ^12^, ^13^, ^14^, ^15^ ^]^ positioning m6Am as a potential therapeutic target in these disorders.

Renal cell carcinoma (RCC) ranks among the most common malignancies in the urinary system, with its incidence rate steadily rising over recent decades. In 2022, the estimated number of new cases and deaths from RCC reached 79,000 and 13,920, respectively.^[^ ^16^ ^]^ 20–30% of the patient presenting with metastasis at their initial diagnosis.^[^ ^17^ ^]^ Distinctively characterized by the high frequency of the inactivating mutation in the tumor suppressor gene Von Hippel‐Lindau (VHL), RCC progression is often driven by the accumulation of hypoxia‐inducible factor (HIF).^[^ ^18^ ^]^ While clinical trials have highlighted HIF as a potential target in cases where first‐line tyrosine kinase inhibitors (TKI) treatment is proved inefficiency, resistance to HIF antagonism persists in a subset of tumors.^[^ ^19^ ^]^

Although RNA modifications have gained attention in RCC, including the downregulation of methyltransferase 14 (METTL14), which leads to cancer metastasis via glycolytic reprogramming,^[^ ^20^ ^]^ and the significant role of FTO in the progression of VHL deficient RCC,^[^ ^21^ ^]^ the function of m6Am in RCC remains unclear, despite its prevalence in 30% of the starting nucleotide in mRNA.

Phosphatidic acid (PA) serves as a crucial precursor for various other phospholipids, constituting the simplest type of glycerophospholipid.^[^ ^22^ ^]^ As a component of the membrane, PA can originate from three main sources: de novo synthesis, hydrolysis of phosphatidylcholine, and phosphorylation of diacylglycerol (DAG).^[^ ^22^ ^]^ Lipid phosphate phosphatases (LPPs) are a group of enzymes that catalyze the dephosphorylation of lipid phosphates, including PA, and subsequently yield DAG.^[^ ^23^ ^]^ This reaction is pivotal for maintaining a balanced synthesis of phospholipid and triacylglycerol.^[^ ^24^ ^]^ Recent studies have shed light on the significance of mitochondrial PA, with research indicating its regulatory role in mitochondrial morphology via regulating both the fusion and fission process.^[^ ^25^, ^26^ ^]^ Despite the burgeoning research on mitochondria in cancer cells,^[^ ^27^ ^]^ the specific impact of PA on mitochondrial morphology and function in the context of cancer remains largely unexplored.

In this study, we identified a significant upregulation in the levels of both PCIF1 and m6Am modification in RCC tissues. Elevated expression of PCIF1 correlates with poor prognosis in RCC patients. Furthermore, we demonstrated that PCIF1 enhances RCC cancer cell proliferation and migration both in vitro and in vivo, with its activity dependent on m6Am catalysis. Mechanically, we uncovered LPP3 as a key target of PCIF1. Through its enhanced translation of the LPP3 protein, PCIF1 promotes oncogenesis by regulating PA level within mitochondria. This regulation, in turn, facilitates mitochondrial fission and sustains a pro‐tumor mitochondrial morphology and function.

## 2 Results

### 2.1 PCIF1 is Highly Expressed in Renal Cell Carcinoma and is Associated with Poor Prognosis

**Figure 1:**
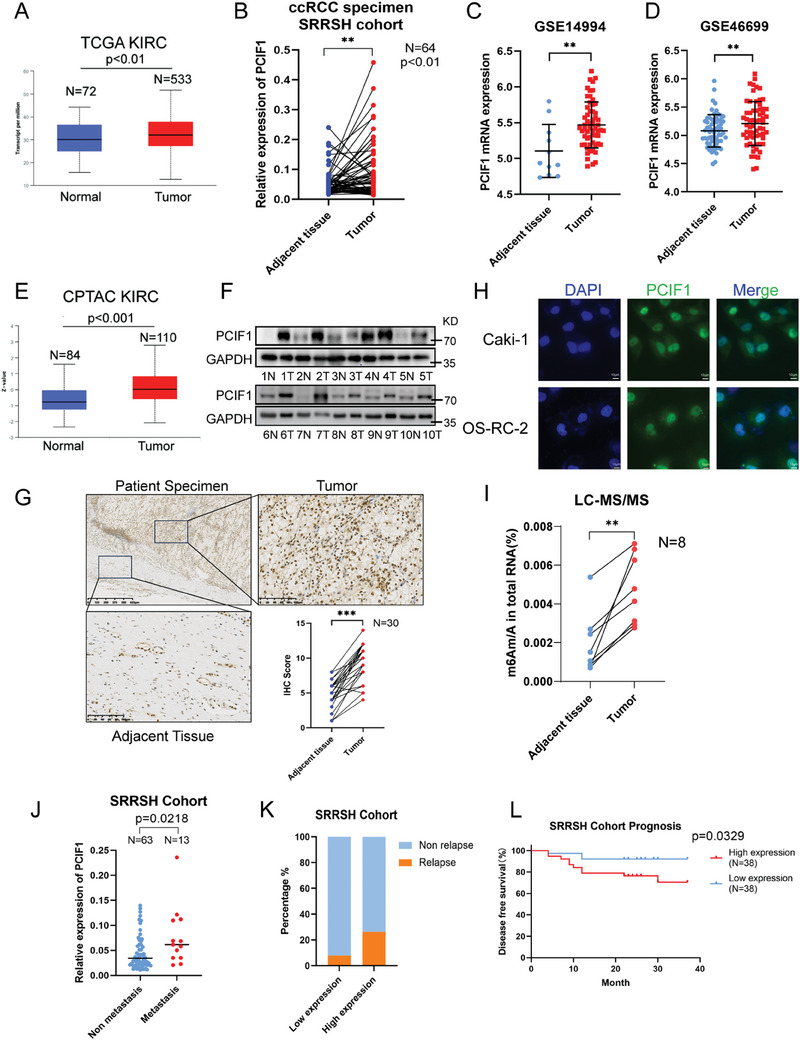
PCIF1 expression is significantly increased in RCC and associated with poor prognosis. A) The expression of PCIF1 in kidney renal cell carcinoma (KIRC) was analyzed with the TCGA database. B) The qRT‐PCR assay results show PCIF1 expression at the mRNA level in RCC specimens and adjacent normal tissues from the SRRSH cohort. C,D) PCIF1 expression in normal tissues and RCC specimens in GSE14994 C) and GSE46699 D) datasets. Data are shown as the mean ± SD. E) The protein expression of PCIF1 in RCC specimens and adjacent normal tissues in the CPTAC database. F) The western blot assays exhibited the PCIF1 protein expression in paired RCC tumor and normal tissues from the SRRSH RCC cohort. G) Representative IHC staining images for PCIF1 protein in the SRRSH RCC cohort are presented. IHC scores are calculated and analyzed. H) Representative immunofluorescence images in Caki‐1 and OS‐RC‐2 cells stained with anti‐PCIF1. Nuclei were stained with DAPI. Scale bar, 10 µm. I) LC‐MS/MS quantification of the m6Am/A ratios in RNAs extracted from 8 pairs of RCC specimens and their corresponding normal tissues. J) Relative PCIF1 expression of SRRSH RCC cohort with or without metastasis. K) The frequency of relapse in RCC patients with low and high expression of PCIF1, the low and high PCIF1 expression groups were cut off by the median expression. L) Kaplan–Meier survival curves of low and high PCIF1 expression groups. ^*^ *P* < 0.05, ^**^ *P* < 0.01, ^***^ *P* < 0.001; ns, not significant.

To explore the clinical relevance of m6Am writer PCIF1 in RCC, we conducted comprehensive gene expression analyses utilizing multiple databases. First, we examined the expression of PCIF1 in the Cancer Genome Atlas (TCGA) RCC cohort, revealing a statistically significant increase of PCIF1 expression in RCC specimens compared to adjacent normal tissues (**Figure** **1**A). The upregulation was validated in our internal SRRSH RCC cohort by qRT‐PCR (Figure 1B) and two RCC microarray analysis^[^ ^28^, ^29^ ^]^ from GEO (Figure 1C,D). Additionally, querying the Clinical Proteomic Tumor Analysis Consortium (CPTAC) Kidney renal clear cell carcinoma (KIRC) cohort demonstrated marked upregulation of PCIF1 at protein level in KIRC tissues (Figure 1E), consistent with higher protein levels detected in RCC specimens from our internal cohort via western blot analysis (Figure 1F). Immunohistochemistry (IHC) further confirmed the overexpression of PCIF1 protein in RCC cells, predominantly localized within the nucleus (Figure 1G). Immunofluorescence staining of RCC cell lines, Caki‐1 and OS‐RC‐2, validated the nuclear localization of PCIF1, underlying its critical role in nascent mRNA 5’ untranslated region (5'‐UTR) processing as a m6Am writer (Figure 1H). To determine whether the m6Am modification level is correspondingly elevated in RCC, we performed liquid chromatography‐tandem mass spectrometry (LC‐MS/MS) analysis on tissue RNA. The result revealed a uniformly upregulated level of m6Am/A in RCC tumors compared to adjacent normal tissues (Figure 1I), in concordance with the increased expression of PCIF1 in RCC.

The significant upregulation of PCIF1 expression and m6Am level in RCC motivated us to investigate the correlation between PCIF1 expression and clinical parameters. Analysis of clinical data from SRRSH cohorts identified a higher expression of PCIF1 in tumors with distant metastasis (Figure 1J). Importantly, patients with higher expression of PCIF1 exhibited a higher frequency of relapse and worse prognosis (Figure 1K,L). Taken together, our findings suggest PCIF1 possesses great potential as a novel predictor in RCC prognosis.

### 2.2 PCIF1 is Crucial to RCC Progression

**Figure 2:**
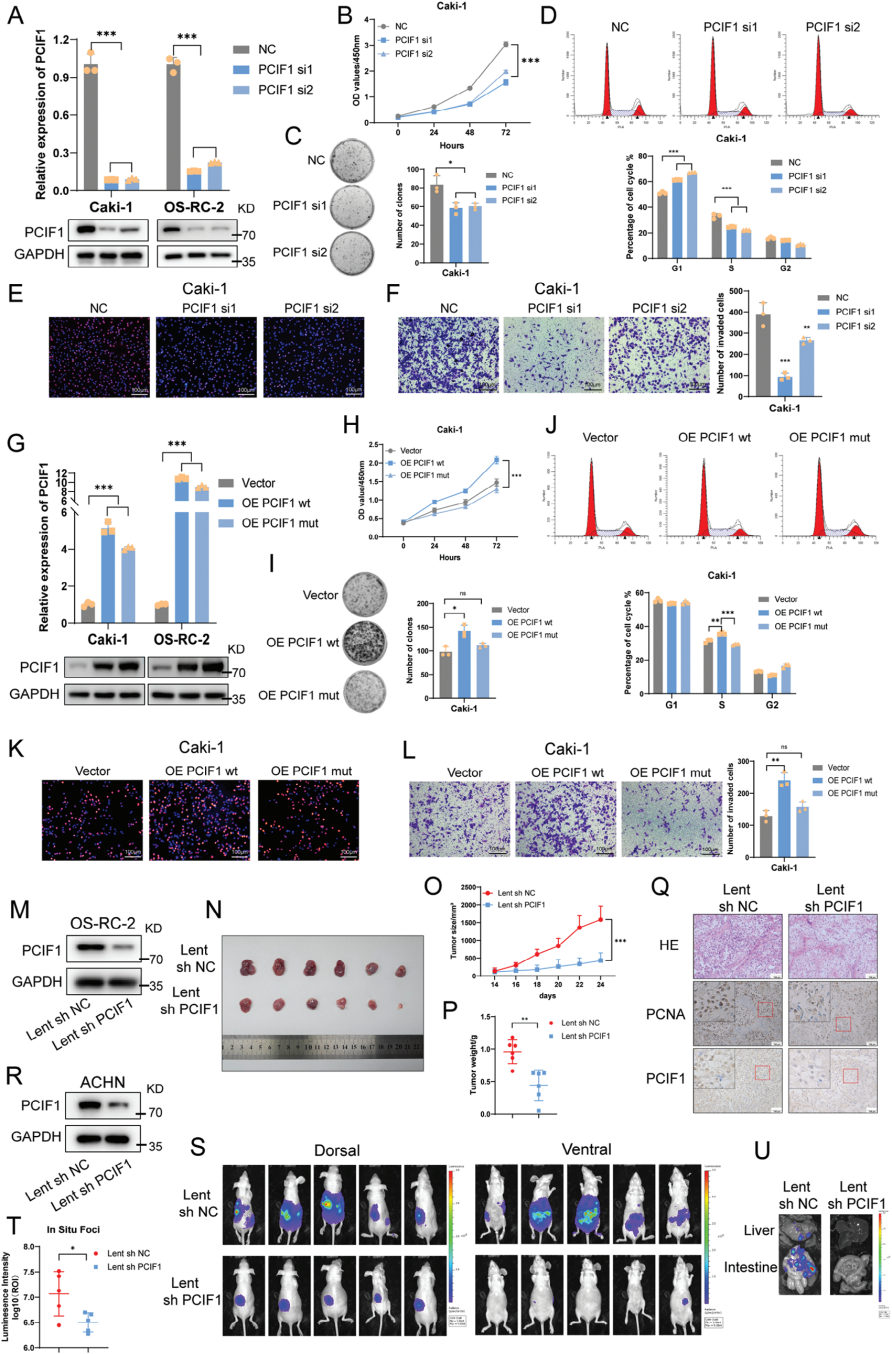
PCIF1 is required for RCC progression in vitro and in vivo. A) qRT‐PCR and western blotting confirmed the knockdown of PCIF1 in Caki‐1 and OS‐RC‐2 cells. B) Optical density at 450 nm (OD450) values of Caki‐1 cells transfected with control or PCIF1 siRNAs in CCK‐8 assay. C) Representative images of colony‐formation assay and its quantification data of indicated Caki‐1 cells. D) Flow cytometric analysis of cell cycle in Caki‐1 cells transfected with control or PCIF1 siRNAs. E) Representative images of EdU assay in indicated Caki‐1 cells. Scale bar, 100 µm. F) Representative images of transwell assay in indicated Caki‐1 cells. Scale bar, 100 µm. The migrated cells are counted and analyzed. G) qRT‐PCR and western blotting showing the overexpression of wild‐type or mutant PCIF1 (N553A) in Caki‐1 and OS‐RC‐2 cells. H) Optical density at 450 nm (OD450) values of Caki‐1 cells overexpressed with vector or wild‐type/mutant PCIF1 in CCK‐8 assay. I) Representative images of colony‐formation assay and its quantification data of indicated Caki‐1 cells. J) Flow cytometric analysis of cell cycle in Caki‐1 cells transfected with vector or wild‐type/mutant PCIF1. K) Representative images of EdU assay in indicated Caki‐1 cells. Scale bar, 100 µm. L) Representative images of transwell assay in indicated Caki‐1 cells. Scale bar, 100 µm. The migrated cells are counted and analyzed. M) Western blotting showing the PCIF1 depletion in OS‐RC‐2 cells with lentivirus‐based control or shRNA. N–P) Images N), volumes O), and weights P) of indicated OS‐RC‐2 cell‐derived xenograft tumors (n = 6). Q) Representative H&E and IHC staining images for PCIF1, PCNA of OS‐RC‐2 cell‐derived xenograft tumors. Scale bar, 100 µm. R) Western blotting showing the PCIF1 depletion in ACHN cells with lentivirus‐based control or shRNA. S,T) Bioluminescent images showing primary foci and metastasis in mice underwent luciferase labeled ACHN injection under renal capsule (n = 5) S). Bioluminescent signal intensities (photons/s/cm2/sr) of primary foci were quantified T). U) Representative bioluminescent images of metastases in the liver and intestine. ^*^ *P* < 0.05, ^**^ *P* < 0.01, ^***^ *P* < 0.001; ns, not significant.

To delve into the impact of PCIF1 on RCC progression, we knocked down PCIF1 in Caki‐1 and OS‐RC‐2 with siRNAs (**Figure** **2**A). Subsequent analysis via cell counting kit‐8 (CCK‐8) assay revealed a significant reduction in RCC cell proliferation (Figure 2B; Figure S1A, Supporting Information). In addition, the colony‐formation ability of these cells was markedly reduced (Figure 2C; Figure S1B, Supporting Information). Flow cytometric analysis demonstrated a decrease in the percentage of cells in the S phase accompanied by an increase in the G1 phase after silencing of PCIF1 (Figure 2D; Figure S1C, Supporting Information), with 5‐ethynyl‐20‐deoxyuridine (EdU) incorporation assay showing suppressed DNA replication activity (Figure 2E; Figure S1D, Supporting Information). Furthermore, the impediment to RCC migration upon PCIF1 depletion was revealed in transwell assays and wound healing assays (Figure 2F; Figure S1E–H, Supporting Information).

To determine whether PCIF1's oncogenic role in RCC progression is reliant on its function as the m6Am methyltransferase, we constructed a catalytically incompetent PCIF1 overexpression plasmid with a N553A mutation^[^ ^9^ ^]^ (Figure 2G). While ectopic expression of wild‐type PCIF1 significantly promoted cell proliferation, colony‐formation, and migration in RCC cells, transfection of N553A mutant PCIF1 yielded minimal alterations (Figure 2H–L; Figure S1I–N, Supporting Information). These results substantiated the importance of PCIF1's methyltransferase activity in RCC progression.

Given our findings, we turned to in vivo models to probe PCIF1's role in RCC progression. We performed subcutaneously cancer cell injection in nude mice, revealing that PCIF1 silencing significantly inhibits tumor growth (Figure 2M–P), corroborated by reduced staining of the proliferation index marker PCNA (Figure 2Q; Figure S1O, Supporting Information). Conversely, stable PCIF1 overexpression showed an opposite effect on tumor growth (Figure S1P–S, Supporting Information). To assess PCIF1's role in tumor metastasis in vivo, we orthotopically injected luciferase‐labeled ACHN RCC cells in nude mice (Figure 2R). While signals of in situ foci decreased due to interfered tumor growth (Figure 2S,T), tumor metastasis was nearly eradicated in the PCIF1 knockdown group, highlighting and direct impact of PCIF1 depletion on RCC metastasis (Figure 2S,U). Taken together, these data underscore PCIF1's critical functions as an oncogene in tumor progression.

### 2.3 LPP3 Emerges as a Key Target of PCIF1 in RCC

**Figure 3:**
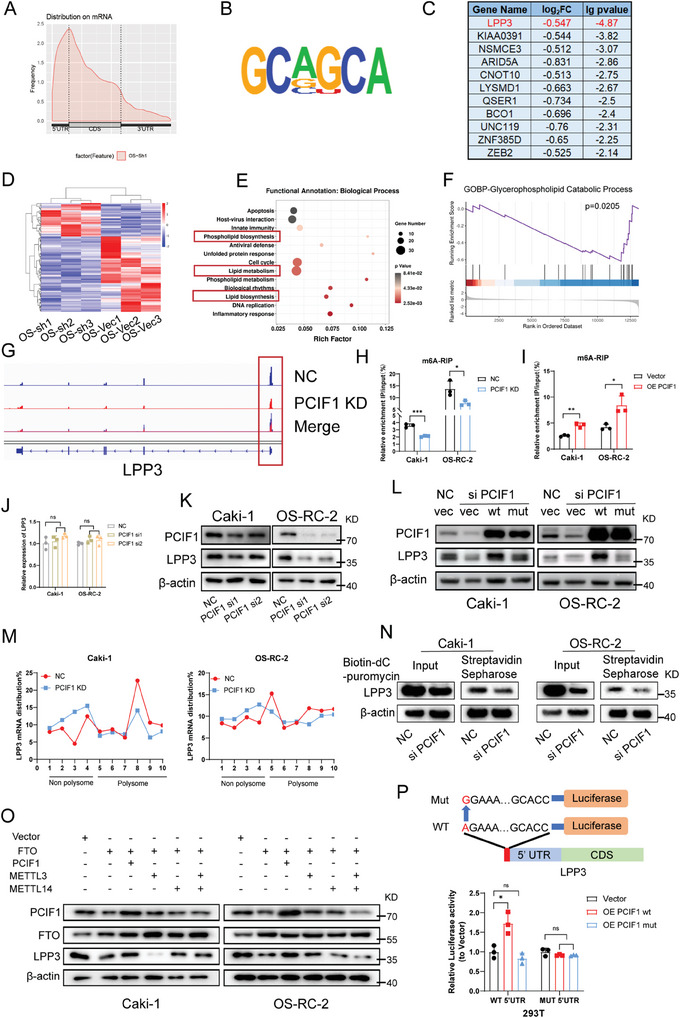
LPP3 was identified as a downstream target of PCIF1. A) Metageneplot showing the distribution of m6Am peaks across mRNA. B) Consensus motif of m6Am peaks presented by HOMER. C) List of genes with differentially exhibited 5'‐UTR m6Am peaks upon PCIF1 knockdown in OS‐RC‐2 cells, log2FC < −0.5, p < 0.01. D) Heatmap showing the mRNA expression change in OS‐RC‐2 upon PCIF1 knockdown. E) Bubble diagram showing the biological processes enrichment of differentially expressed genes upon PCIF1 knockdown. F) Gene set enrichment analysis of Glycerophospholipid Catabolic Process pathway upon PCIF1 knockdown. G) Integrative Genomics Viewer (IGV) tracks displaying m6Am peaks on LPP3 in control and PCIF1‐depleted OS‐RC‐2 cells. H,I) m6A‐RIP assay showing the m6A/m6Am level on LPP3 in RCC cells with the silencing H) or overexpression I) of PCIF1. J) qRT‐PCR assay showing the mRNA level of LPP3 in RCC cells transfected with indicated PCIF1 siRNAs. K,L) Western blotting assay showing the protein level of LPP3 in RCC cells transfected with indicated PCIF1 siRNAs and plasmids. M) qRT‐PCR assay showing the distribution of LPP3 mRNA in different polysome gradient fractions in control and PCIF1‐depleted RCC cells. N) Western blotting assay showing the effect of PCIF1 knockdown on nascent LPP3 labeled with biotin‐dC‐puromycin. O) Western blotting showing the protein level of LPP3 in RCC cells transfected with indicated plasmids. P) Schematic diagram of LPP3 5′‐UTR WT and 5′‐UTR MUT firefly luciferase reporters. Relative luciferase activity in 293T cells transfected with firefly luciferase reporters, renilla luciferase vector, and indicated PCIF1 overexpression plasmids. ^*^ *P* < 0.05, ^**^ *P* < 0.01, ^***^ *P* < 0.001; ns, not significant.

To identify the target genes of PCIF1, we conducted m6Am‐exo‐Seq in RCC cells.^[^ ^11^ ^]^ Metagene plot analyses of peaks showed an enrichment in 5′‐UTR, deviating from the typical m6A distribution around stop codon regions (**Figure** **3**A; Figure S2A, Supporting Information). Analysis of the peaks unveiled a characteristic CA motif^[^ ^10^, ^30^ ^]^ (Figure 3B). Potential target genes were pinpointed based on fold change and p‐value (log_2_FC < −0.5, p < 0.01) (Figure 3C; Figure S2B,C, Supporting Information). To discern the key downstream targets of PCIF1, we conducted functional enrichment analysis with differentially expressed genes from the input samples. This analysis highlighted several biological processes, such as “lipid metabolism”, “lipid biosynthesis” and “phospholipid metabolism” (Figure 3D,E). GSEA analysis indicated significant impacts on glycerophospholipid and glycerolipid metabolism upon PCIF1 silencing (Figure 3F; Figure S2D, Supporting Information). Notably, phospholipid phosphatase 3 (LPP3), an enzyme catalyzing the dephosphorylation of glycerolipid and sphingolipid phosphate esters, is at the top of our downstream target candidates (Figure 3C).

Integrative Genomics View plots of m6Am‐exo‐Seq data displayed a reduced peak around transcription start site (TSS) of the LPP3 transcript after PCIF1 knockdown (Figure 3G). To confirm PCIF1's regulation of m6Am modification on LPP3, we further employed RNA immunoprecipitation (RIP)‐qPCR assay with anti‐m6A antibody. Results demonstrated decreased m6A/m6Am level on the LPP3 transcript upon PCIF1 depletion in Caki‐1 and OS‐RC‐2 cells (Figure 3H), with the opposite effect observed upon PCIF1 overexpression (Figure 3I). These findings confirm the PCIF1 catalyzing m6Am site on the TSS of LPP3 transcript.

Subsequently, we sought to explore the effect of m6Am modification on LPP3. LPP3 mRNA level and its stability remain unaffected by PCIF1 regulation in RCC cells (Figure 3J; Figure S2E,F, Supporting Information). However, western blot analysis revealed markedly suppressed LPP3 protein expression in the PCIF1 knockdown group (Figure 3K). Reciprocally, ectopic expression of wild‐type PCIF1 but not the catalytic incompetent mutant significantly reversed the reduction of LPP3 protein abundance caused by PCIF1 depletion (Figure 3L). Minimal difference in cycloheximide (CHX) chase assay ruled out the direct or indirect effect of PCIF1 on protein stability (Figure S2G, Supporting Information). To examine whether PCIF1 regulates LPP3 protein translation, we conducted polysome profiling analysis and observed significantly decreased polysome occupancy on LPP3 mRNA in PCIF1 knockdown groups (Figure 3M). Additionally, the puromycin labeling assay revealed a reduction in labeled nascent LPP3 upon PCIF1 depletion (Figure 3N). These results indicate that PCIF1 knockdown inhibits LPP3 translation. Interestingly, a decreased polysome portion of global RNA was also observed, suggesting a broad impact of PCIF1 in promoting mRNA translation in RCC, which is further confirmed by puromycin intake assay (Figure S2H,I, Supporting Information). FTO has been recognized as the eraser of both m6A and m6Am.^[^ ^31^ ^]^ Overexpression of FTO led to a remarkable downregulation of LPP3 protein expression, which can be rescued by PCIF1 overexpression, but not by m6A methyltransferases METTL3 and/or METTL14 (Figure 3O). To further validate the regulatory role of m6Am on LPP3 translation, a dual luciferase reporter assay was conducted. The results showed that wild‐type PCIF1 could only enhance the translational efficiency of the reporter containing the m6Am‐modifiable site from the 5′‐UTR of LPP3 mRNA, while its catalytical incompetent mutant had minimum effect on luminescence with both reporters (Figure 3P). Altogether, these findings demonstrated that PCIF1 promotes the translational efficiency of LPP3 mRNA by depositing m6Am on its starting site.

### 2.4 LPP3 Mediates the Oncogenic Role of PCIF1 in RCC

**Figure 4:**
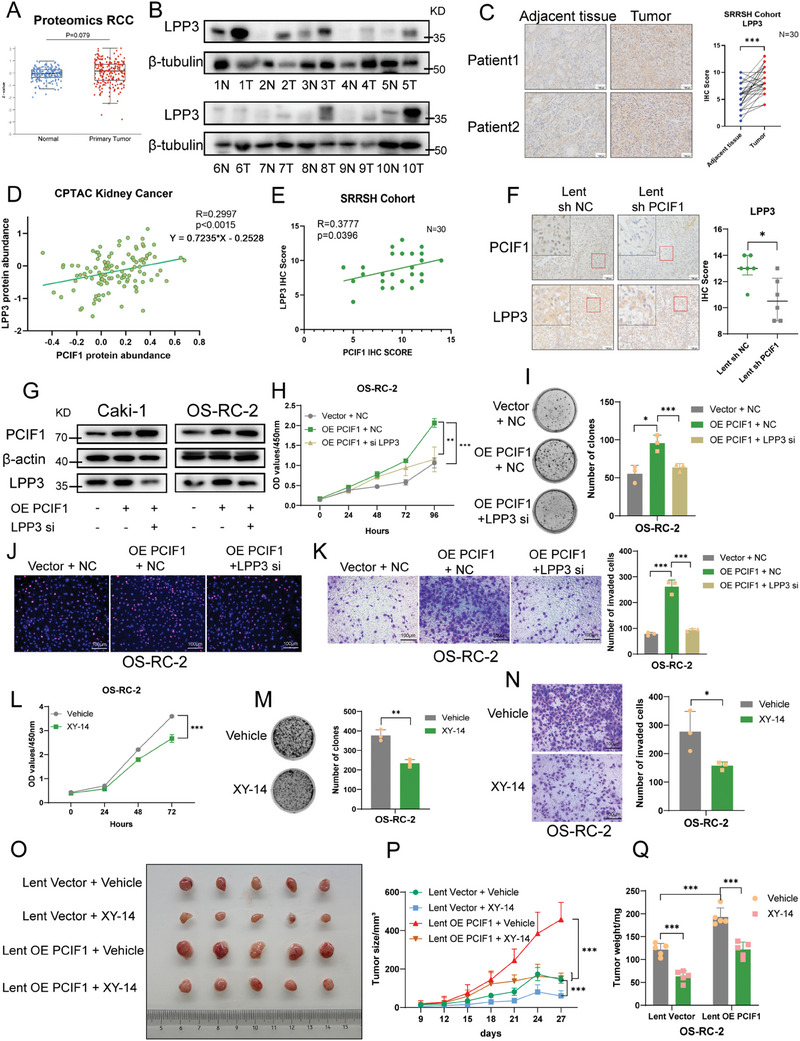
LPP3 mediated the oncogenic role of PCIF1 in RCC progression. A) The protein expression of LPP3 in RCC specimens and adjacent normal tissues in the CPTAC database. B) The western blotting shows the LPP3 protein expression in paired RCC tumor and normal tissues from the SRRSH RCC cohort. C) Representative IHC staining images for LPP3 protein in the SRRSH RCC cohort. Scale bar, 100 µm. IHC scores are calculated and analyzed. D,E) Pearson's correlation analysis shows a positive correlation between PCIF1 and LPP3 expression in proteomics data from the CPTAC RCC dataset (D, n = 110) and IHC scores of the SRRSH RCC cohort (E, n = 30). F) Representative IHC staining images for PCIF1 and LPP3 protein of OS‐RC‐2 cell‐derived xenograft tumors. Scale bar, 100 µm. IHC scores are calculated and analyzed. G) Western blotting showing the protein level of LPP3 in RCC cells transfected with indicated plasmids and siRNAs. H) Optical density at 450 nm (OD450) values of OS‐RC‐2 cells transfected with indicated plasmids and siRNAs in CCK‐8 assay. I) Representative images of colony‐formation assay and its quantification data of indicated OS‐RC‐2 cells. J) Representative images of EdU assay of indicated OS‐RC‐2 cells. Scale bar, 100 µm. K) Representative images of transwell assay in OS‐RC‐2 cells transfected indicated plasmids and siRNAs. Scale bar, 100 µm. The migrated cells are counted and analyzed. L) Optical density at 450 nm (OD450) values of OS‐RC‐2 cells treated with vehicle or XY‐14 (10µM) in cell counting kit‐8 (CCK‐8) assay. M) Representative images of colony‐formation assay and its quantification data of OS‐RC‐2 cells treated with vehicle or XY‐14 (10 µM). N) Representative images of transwell assay in OS‐RC‐2 cells treated with vehicle or XY‐14 (10 µM). Scale bar, 100 µm. The migrated cells are counted and analyzed. (O‐Q) Images O), volumes P), and weights Q) of cell‐derived tumors from control or PCIF1 overexpressed OS‐RC‐2 cells treated with vehicle or XY‐14 (n = 5). ^*^ *P* < 0.05, ^**^ *P* < 0.01, ^***^ *P* < 0.001; ns, not significant.

Having identified LPP3 as a key target of PCIF1, we further set to explore its role in the oncogenic effect of PCIF1 in RCC progression. Initial investigation into the CPTAC RCC dataset revealed a trend of higher LPP3 abundance in tumor tissue compared to adjacent normal tissue, although with a p‐value slightly beyond the statistical criterion (**Figure** **4**A). Subsequent western blot and IHC assay in our internal cohort confirmed the increased protein level of LPP3 in RCC (Figure 4B,C). Moreover, a positive correlation between PCIF1 and LPP3 at the protein level was disclosed both in CPTAC RCC proteomics data and IHC staining scores from our internal RCC samples (Figure 4D,E). Additionally, decreased IHC staining of LPP3 was noted in the subcutaneous tumors from the PCIF1 knockdown group compared to control ones (Figure 4F).

To probe the function of LPP3 in RCC development, we knocked down LPP3 (Figure S3A, Supporting Information), resulting in significant impairment of RCC proliferation and migration (Figure S3B–E, Supporting Information). Conversely, LPP3 overexpression intensified the aggressiveness of RCC cancer cells (Figure S3F–I, Supporting Information). Rescue experiments further demonstrated that LPP3 knockdown abolished the oncogenic effect of PCIF1 in RCC (Figure 4G–K; Figure S3J–M, Supporting Information). Notably compound XY‐14, reported to competitively inhibit lipid phosphate phosphatase,^[^ ^32^ ^]^ effectively restrained RCC cell proliferation and migration (Figure 4L–N; Figure S3N–P, Supporting Information). Furthermore, the in vivo subcutaneous tumor implantation model showed that intratumor injection of XY‐14 efficiently counteracted the stimulative effect of PCIF1 overexpression on tumor growth (Figure 4O–Q), highlighting the potential of targeting LPP3 with its inhibitor in RCC treatment. In summary, these results established LPP3 as a key downstream target of PCIF1, facilitating RCC progression.

### 2.5 LPP3 is Essential to Maintain Phosphatidic Acid Balance in Mitochondria

**Figure 5:**
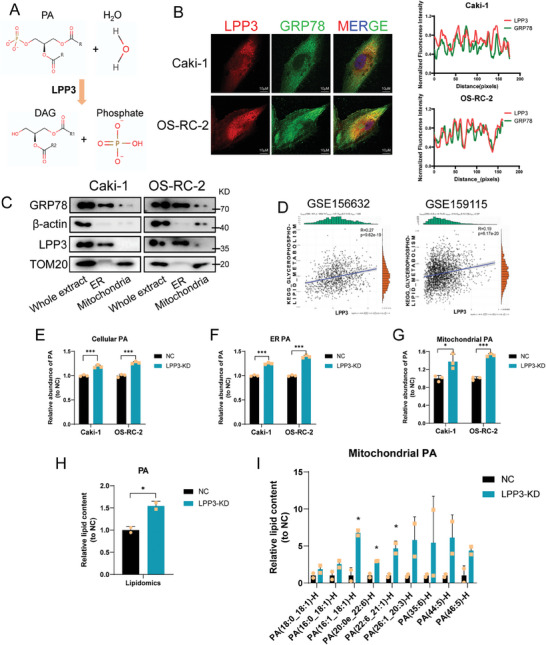
LPP3 knockdown led to phosphatidic acid piling up in mitochondria. A) Schematic diagram showing LPP3 catalyzes the hydrolysis of phosphatidic acid. B) Representative immunofluorescence images in Caki‐1 and OS‐RC‐2 cells stained with anti‐LPP3, anti‐GRP78, and DAPI. Scale bar, 10 µm. C) Western blotting of LPP3 and different markers in the endoplasmic reticulum and mitochondria fractions of RCC cells. D) Correlation of LPP3 expression and KEGG Glycerophospholipid Metabolism enrichment scores in malignant cells in two RCC tumor single‐cell datasets GSE156632 and GSE159115. (E‐G) The Elisa assay shows the level of cellular E), endoplasmic reticular F), and mitochondrial G) PA. Data are representative of three independent experiments and are shown as the mean ± SD. H) Quantification of mitochondrial PA level of control and PCIF1‐knockdown Caki‐1 cells, relative to control condition. I) Quantification of different PA subclasses levels in mitochondria of control and PCIF1‐knockdown Caki‐1 cells, relative to control condition. Data are representative of two independent experiments and are shown as the mean ± SD. ^*^ *P* < 0.05, ^**^ *P* < 0.01, ^***^ *P* < 0.001; ns, not significant.

LPP3, also known as type 2 phosphatidic acid phosphatase β (PPAP2B), functions as a magnesium‐independent phospholipid phosphatase capable of catalyzing the hydrolysis of phospholipids, including phosphatidate/PA, lysophosphatidate/LPA, sphingosine 1‐phosphate/S1P and ceramide 1‐phosphate/C1P (**Figure** **5**A).^[^ ^23^, ^33^ ^]^ As an integral membrane protein, previous studies have demonstrated that LPP3 exhibits cell‐specific subcellular localization, including the plasma membrane, endoplasmic reticulum (ER) membrane, and Golgi complex.^[^ ^34^, ^35^, ^36^ ^]^ In RCC cells, our immunofluorescence assay showed the minimal distribution of LPP3 on the plasma membrane and Golgi complex but revealed an obvious colocalization with the ER marker Glucose‐Regulated Protein 78 (GRP78) (Figure 5B; Figure S4A,B, Supporting Information). In addition, western blot analysis of cellular fractions exhibited LPP3 was enriched in ER fraction (Figure 5C). These data indicate that ER, a dynamic organelle crucial for various biological processes, including protein synthesis, calcium storage, and lipid synthesis,^[^ ^37^ ^]^ serves as the primary site for LPP3 in RCC cells. In alignment with the ER's central role in lipid metabolism, a significant association between LPP3 expression and the enrichment score of KEGG_Glycerophospholipid_Metabolism in cancer cells was observed in two published RCC single‐cell datasets (Figure 5D).^[^ ^38^, ^39^ ^]^ Given the high affinity of LPP3 for PA among all phospholipid substrates,^[^ ^33^ ^]^ we questioned whether LPP3 could regulate the PA level in RCC cells. ELISA assay revealed an accumulation of PA in both ER fractions and whole‐cell extracts from Caki‐1 and OS‐RC‐2 upon LPP3 depletion (Figure 5E,F). Notably, there is abundant lipid transfer between ER and mitochondria at mitochondria‐associated ER membranes (MAMs).^[^ ^40^ ^]^ PA in mitochondria, recently disclosed as a significant regulator of mitochondrial morphology and function, can either be generated locally or imported from the ER.^[^ ^41^, ^42^, ^43^ ^]^ Therefore, we sought to investigate whether LPP3 affects PA content in mitochondria. Lipidomics analysis and ELISA demonstrated that LPP3 depletion led to increased PA levels in mitochondrial fractions extracted from RCC cells (Figure 5G–I). Furthermore, mitochondrial PA can be converted into diacylglycerol (DAG) and cardiolipin (CL), with phosphatidylglycerol (PG) serving as an intermediate^[^ ^24^ ^]^ (Figure S4C, Supporting Information). Our lipidomics results revealed an elevated level of mitochondrial DAG and CL correspondingly upon LPP3 knockdown (Figure S4D,E, Supporting Information), while PG remains stable (Figure S4F, Supporting Information), consistent with previous findings indicating no PG accumulation under normal conditions.^[^ ^44^ ^]^ These findings collectively demonstrate that LPP3 depletion leads to an accumulation of PA in mitochondria.

### 2.6 PCIF1‐LPP3 Axis Promotes Mitochondrial Fission and Fuels OXPHOS in RCC

**Figure 6:**
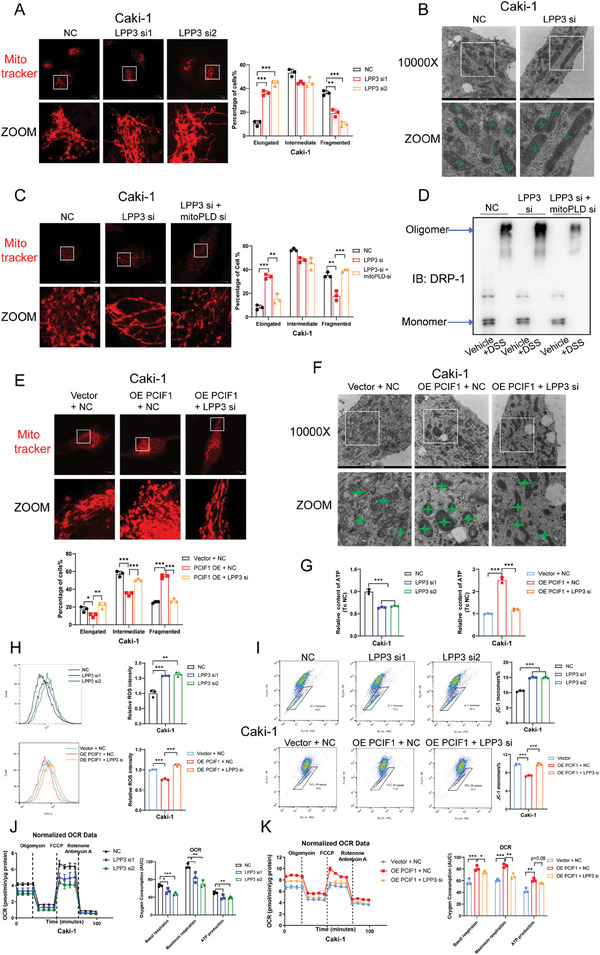
PCIF1/LPP3 axis facilitated mitochondrial fission and enhanced mitochondrial respiration in RCC. A) Representative morphology of mitochondria stained by mitotracker in indicated Caki‐1 cells and quantification of mitochondrial network. Scale bar, 10 µm. Around 50 cells per group were assessed. B) Representative morphology of mitochondria revealed by electron microscopy in indicated Caki‐1 cells. Scale bar, 1 µm. C) Representative morphology of mitochondria stained by mitotracker in Caki‐1 cells transfected with indicated siRNAs and quantification of mitochondrial network. Scale bar, 10 µm. Around 50 cells per group were assessed. D) Western blotting showing the monomers and oligomers of DRP‐1 in Caki‐1 cells transfected with indicated siRNAs. E) Representative morphology of mitochondria stained by mitotracker in indicated Caki‐1 cells with quantification of mitochondrial network. Scale bar, 10 µm. Around 50 cells per group were assessed. F) Representative morphology of mitochondria revealed by electron microscopy in indicated Caki‐1 cells. Scale bar, 1 µm. G) Cellular ATP levels in indicated Caki‐1 cells. H) Flow cytometric analysis showing ROS levels in indicated Caki‐1 cells. The average ROS levels are calculated. I) Flow cytometric analysis showing the mitochondrial membrane potential in indicated Caki‐1 cells. The proportion of cells with JC‐1 monomers was quantified. J,K) Oxygen consumption rate was detected in indicated Caki‐1 cells. Oligomycin, FCCP, rotenone, and antimycin A were added at indicated time points. ^*^ *P* < 0.05, ^**^ *P* < 0.01, ^***^ *P* < 0.001; ns, not significant. Data are representative of three independent experiments and are shown as the mean ± SD.

Multiple studies have reported that mitochondrial PA can hinder its fission and result in mitochondrial elongation.^[^ ^26^, ^45^, ^46^ ^]^ Intriguingly, both fluorescence microscopy and electron microscopy unveiled that LPP3 knockdown led to significantly longer mitochondria in RCC cells (**Figure** **6**A,B; Figure S5A, Supporting Information), without affecting the expression of recognized mitochondrial morphology regulators, such as dynamin‐related protein‐1 (DRP‐1), mitochondrial fission 1 protein (FIS1), mitofusin‐1 (MFN1), mitofusin‐2 (MFN2) and optic atrophy 1 (OPA1) (Figure S5B, Supporting Information). To further confirm that this elongation was due to mitochondrial PA accumulation, we knocked down mitoPLD, the enzyme responsible for PA generation in mitochondria, to counteract the enhanced import of PA from the ER upon LPP3 depletion. Remarkably, inhibition of PA generation in mitochondria rescued the elongated morphology induced by LPP3 knockdown (Figure 6C). PA has been identified to restrain mitochondrial fission via binding to DRP‐1, inhibiting its oligomerization‐stimulated GTP hydrolysis and consequent membrane constriction, accompanied with enhanced ineffective oligomerization of DRP‐1.^[^ ^26^ ^]^ Consistently, as DRP‐1 protein level remains stable upon LPP3 depletion (Figure S5B, Supporting Information), we observed an increase in DRP‐1 oligomer level, which was attenuated after eliminating accumulated PA via mitoPLD knockdown (Figure 6D; Figure S5C, Supporting Information). In addition, immunofluorescence staining revealed larger DRP‐1 foci in LPP3 silencing RCC cells, which were restored by mitoPLD depletion (Figure S5D, Supporting Information).

We then questioned whether the oncogenic PCIF1/LPP3 axis can regulate mitochondrial morphology in RCC cells. Using fluorescence microscopy and electron microscopy, we found PCIF1 overexpression resulted in more fragmented mitochondria, and LPP3 depletion can markedly reverse this trend (Figure 6E,F; Figure S5E, Supporting Information). Likewise, the expression of classical mitochondrial dynamics regulators remained unaffected (Figure S5F, Supporting Information).

Mitochondrial morphology is closely connected with its bioenergetics.^[^ ^47^ ^]^ We found that LPP3 knockdown reduced cellular ATP levels and reversed the increase of ATP induced by PCIF1 ectopic expression in RCC cells (Figure 6G; Figure S5G, Supporting Information). Furthermore, mitochondrial dysfunction was evident upon LPP3 knockdown, reflected in increased reactive oxygen species (ROS) levels and decreased mitochondrial membrane potential (MMP) (Figure 6H,I; Figure S5H,I, Supporting Information). Conversely, PCIF1 overexpression optimized mitochondrial function in RCC cells, reducing ROS levels and enhancing MMP, effects that were completely abrogated by LPP3 silencing (Figure 6H,I; Figure S5H,I, Supporting Information). Moreover, seahorse assays indicated that targeting LPP3 inhibited the basal oxidative phosphorylation (OXPHOS) level and mitochondrial respiration capacity in RCC cells (Figure 6J). Similarly, PCIF1 distinctly fueled mitochondrial respiration, which was disrupted by blocking the expression of its downstream target LPP3 (Figure 6K). Collectively, our data suggest PCIF1/LPP3 axis regulates mitochondrial morphology and optimizes bioenergetics function to drive RCC progression.

### 2.7 Targeting PCIF1 Enhances Sunitinib Sensitivity in RCC

Tyrosine kinase inhibitors (TKI), such as sunitinib, and pazopanib, are established as the first line therapeutic option for advanced RCC. However, their effectiveness is often hindered by both primary and acquired drug resistance, leading to diminished clinical outcomes. Lately, multiple investigations have shed light on a connection between TKI resistance and a metabolic shift marked by enhanced OXPHOS.^[^ ^48^, ^49^ ^]^ Given that PCIF1 enhances OXPHOS in RCC, we are intrigued by the possibility that suppressing PCIF1 could sensitize RCC cells to sunitinib treatment.

**Figure 7:**
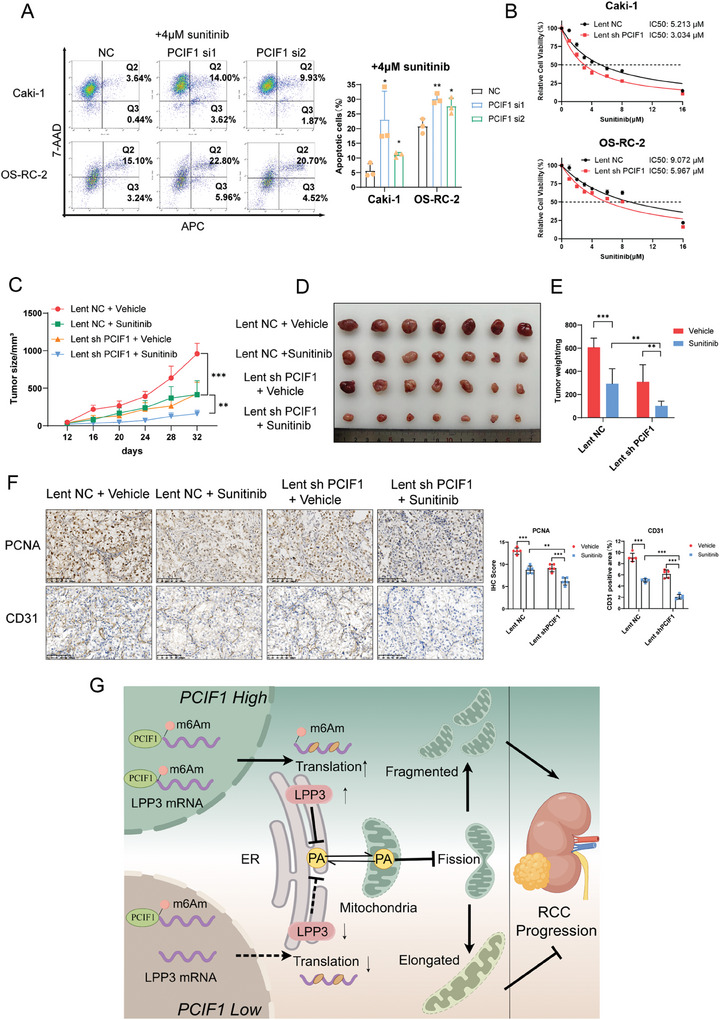
Targeting PCIF1 sensitizes RCC to sunitinib treatment. A) Flow cytometric analysis showing the apoptotic ratio of Caki‐1 and OS‐RC‐2 cells with PCIF1 knockdown under 4 µM sunitinib treatment. B) CCK‐8 assay of control and PCIF1‐knockdown RCC cell lines with sunitinib treatment at indicated concentrations for 48 h. C–E) Volumes C), images D), and weights E) of cell‐derived tumors from control or PCIF1‐knockdown OS‐RC‐2 cells treated with DMSO or sunitinib (40 mg kg^−1^ day^−1^) (n = 7). F) Representative IHC staining images for PCNA, CD31 of OS‐RC‐2 cell‐derived xenograft tumors. Scale bar, 100 µm. IHC score and CD31 positive area are calculated and analyzed (n = 5). G) Diagram of the proposed molecular mechanisms for PCIF1 involvement in RCC progression. ^*^ *P* < 0.05, ^**^ *P* < 0.01, ^***^ *P* < 0.001; ns, not significant.

Interestingly, while the depleting of PCIF1 itself didn't induce an increase in cell apoptosis in Caki‐1 and OS‐RC‐2 (Figure S6A, Supporting Information), RCC cells with PCIF1 knockdown showed an enhanced tendency to undergo cell apoptosis when treated with sunitinib (**Figure** **7**A). Additionally, silencing of PCIF1 notably reduced the half‐maximal inhibitory concentration (IC50) of sunitinib in RCC cell lines (Figure 7B). To validate this effect in vivo, we performed subcutaneous implantation followed by oral treatment of sunitinib in nude mice. Concordantly, while sunitinib treatment could effectively inhibit tumor growth in the control groups, the PCIF1 knockdown group exhibited a superior response to treatment reflected in tumor weight and size (Figure 7C–E), accompanied by a significantly decreased staining of PCNA and angiogenesis marker CD31 (Figure 7F). Collectively, these results indicate inhibition of PCIF1 could enhance the efficacy of sunitinib in RCC.

## 3 Discussion

In 1975, m6Am was initially identified as an RNA modification adjacent to m7G cap structure on mRNA.^[^ ^7^ ^]^ However, only in recent years has significant attention been directed toward elucidating its function and biological significance in disease. The discovery of its eraser, FTO, and writer, PCIF1, has sparked interest in understanding its role further.^[^ ^8^, ^9^ ^]^ Recent studies hinted at its vital involvement in tumor progression. Zhuo et al. demonstrated that PCIF1 promoted the proliferation and invasion of gastric cancer by targeting TM9SF1.^[^ ^12^ ^]^ In colorectal cancer, Wang et al. indicated PCIF1 is indispensable for tumorigenesis and can be targeted to enhance the efficacy of immunotherapy.^[^ ^13^ ^]^ A recent study reported that PCIF1 binds to its cofactor CTBT2, contributing jointly to the development of head and neck squamous cell carcinoma.^[^ ^50^ ^]^ Despite these advancements, the role of m6Am in RCC remains poorly understood, even though RCC progression was recognized to be closely linked to epigenetic dysregulation. In our study, we identified overexpression of PCIF1 in RCC, providing evidence to support its essential role in driving tumor progression.

Furthermore, employing m6Am‐exo‐Seq, we elucidated its key downstream functional target as LPP3. Until now, a consensus regarding the functions of m6Am on mRNA has not been reached. While some studies claim m6Am enhances mRNA stability,^[^ ^13^, ^31^ ^]^ others propose its primary influence on mRNA translation.^[^ ^9^, ^11^, ^12^ ^]^ Moreover, opposite perspectives exist on its impacts on translation. In our investigation, we observed a reduction in global translation following PCIF1 depletion. However, we acknowledge the complexity of extending our conclusion to other cells, as varied cellular contexts may yield divergent outcomes. To reconcile these discrepancies across different backgrounds, a thorough understanding of the precise role of m6Am during the mRNA translation process is imperative. Additionally, existing research has disclosed the function of other RNA modifications, such as m6A, heavily depends on the specific “reader” proteins. Thus, identifying the key “reader” proteins responsible for recognizing m6Am represents a critical avenue for future research endeavors.

Renal cell carcinoma has been increasingly recognized as a metabolic disorder characterized by remarkable metabolism reprogramming.^[^ ^51^ ^]^ A crucial aspect of this metabolism reprogramming is the altered lipid metabolism.^[^ ^52^ ^]^ Lipidomics profiles exhibited RCC tissues were distinguished by accumulation of cholesterol esters, and triacylglycerols, alongside a decrease in most phospholipids.^[^ ^53^ ^]^ Previous studies have revealed the significance of certain phospholipids, such as phosphatidylcholine and phosphatidylinositol in the progression of RCC.^[^ ^54^, ^55^ ^]^ Here, we disclosed how phosphatidic acid, the simplest phospholipid, gets involved in this process. By targeting LPP3, the phospholipid phosphatase responsible for PA hydrolysis, we demonstrated an accumulation of PA within mitochondria. In consistency with the established role of mitochondrial PA in repressing mitochondrial fission,^[^ ^26^, ^43^, ^46^ ^]^ we subsequently observed remarkable mitochondrial elongation upon LPP3 depletion. Parallelly, previous research has reported two other enzymes located outside of mitochondria, Lipin 1b in *Drosophila* and PA‐PLA1 in mammalian, also regulate mitochondrial fission via metabolizing PA.^[^ ^56^, ^57^ ^]^ Extensive trafficking of phospholipids from the ER to mitochondria occurs through the sophisticated mitochondria‐associated membrane (MAM) structure, which consists of various tethering proteins and lipid transfer proteins.^[^ ^58^, ^59^ ^]^ Interestingly, mitochondrial fission also takes place adjacent to these contact sites.^[^ ^60^ ^]^ Thus, given our findings that silencing LPP3 results in elevated cellular PA in RCC, future investigation should delve into the precise mechanism by which PA is transferred through MAM and finally deposited at sites of mitochondrial constriction.

Fragmented mitochondria have been recognized as a characteristic in multiple carcinomas, contributing to increased mitochondrial respiration essential for tumor progression.^[^ ^61^, ^62^, ^63^ ^]^ Mitochondrial fission is vital to the quality control of the mitochondrial network, as mitophagy could efficiently degrade fragmented defective mitochondria.^[^ ^64^ ^]^ Excessive elongation of mitochondria is associated with compromised mitochondrial respiratory capacity.^[^ ^65^, ^66^ ^]^ In our study, we comprehensively evaluated the mitochondrial function by measuring ATP content, ROS levels, membrane potential, and OXPHOS levels. Our finding unveiled that the oncogenic PCIF1/LPP3 axis enhances mitochondrial bioenergetics in RCC. While upregulated glycolysis, known as the Warburg effect in tumor cells, has traditionally attracted intensive research attraction in cancer metabolism, the significance of OXPHOS has been overlooked. Until recently, emerging studies have underscored the pivotal role of OXPHOS in certain cancers and presented it as a promising treatment target.^[^ ^67^, ^68^ ^]^ A comprehensive analysis of multi‐omics data from 103 treatment naïve clear cell renal cell carcinoma patients uncovered an upregulation of the OXPHOS pathway in advanced tumors compared to early‐stage tumors.^[^ ^69^ ^]^ Moreover, compelling evidence suggests that impairing RCC mitochondrial respiration effectively impedes tumor progression.^[^ ^70^, ^71^ ^]^ Consistently, we identified how the PCIF1/LPP3 axis modulates mitochondrial respiration in RCC, thereby driving oncogenic effects, and revealed an intricate regulatory interplay involving epigenetics and phospholipid metabolism in energy metabolism (Figure 7G).

## 4 Conclusion

In conclusion, our study identified PCIF1 as a significant epigenetic regulator that promotes the progression of renal cell carcinoma. Mechanically, LPP3 emerges as a pivotal downstream target, with its translation enhanced by PCIF1 through m6Am modification. LPP3 mediated the oncogenic role of PCIF1 by exerting its lipid phosphate phosphatase activity to metabolize phosphatidic acid in RCC, preventing potential PA accumulation in mitochondria. This process facilitates mitochondrial fission and optimizes mitochondrial function, thereby supporting RCC progression.

## 5 Experimental Section

### Human Specimens

RCC specimens and adjacent normal tissues included in the cohort were collected by the department of urology, Sir Run Run Shaw hospital, with approval from the Ethics Committee of SRRSH (20220317). Informed consent was obtained from all patients. The clinical data of patients are provided in Table S1 (Supporting Information).

### Cell Culture and Transfection

Human RCC cell lines OS‐RC‐2, ACHN, and Caki‐1 were purchased from the Cell Bank of Type Culture Collection of the Chinese Academy of Sciences. OS‐RC‐2 was cultured in RPMI‐1640 with 10% FBS (Cellmax, China), ACHN was cultured in MEM medium containing 10% FBS (Cellmax), and the Caki‐1 cell line was cultured in McCoy 5A medium with 10% FBS (Cellmax). All cells were maintained in a 37 °C incubator containing 5% CO_2_. According to the manufacturer's instructions, Polyplus jetPRIME (Bestopbio) was used for plasmids and siRNAs transfection. siRNAs were synthesized by Genepharma (China) with all sequences displayed in Table S2 (Supporting Information), plasmids were constructed and produced by GeneChem (China). For stable transfection, the lentivirus was designed, synthesized, and collected by GENECHEM (China) and used to infect ccRCC cells with a transfection reagent provided by GeneChem (China). Stable infected cell lines were subsequently selected using puromycin (Selleck).

### Animal Experiment

All procedures in the in vivo experiment conformed to the institutional guidelines and were approved by the Animal Research Ethics Committee of Zhejiang University (SRRSH202208083). For the subcutaneous implantation model, 1 × 10^6^ OS‐RC‐2 cells were suspended in a 50 µL mixture of PBS and Matrigel (Corning) with a 1:1 ratio, and subsequently subcutaneously injected into 4 weeks BALB/C Nude mice. Tumor volumes were measured daily with the formula: Volume = (Width^2^ × Length)/2. For sunitinib (Selleck) treatment, mice were orally treated with vehicle or sunitinib (40 mg kg^−1^ day^−1^). For XY‐14 (Echelon) treatment, intratumoral injections of either the vehicle or XY‐14 (0.1 mM, 50 µL) were administered every other day. For the renal orthotopic implantation model, 2 × 10^6^ ACHN cells were suspended in a 50 µL mixture of PBS and Matrigel with a 1:1 ratio and subsequently injected under the renal capsule of 4 weeks BALB/C Nude mice. After 8 weeks, mice were anesthetized and an in vivo imaging system (IVIS) was used to detect tumor growth and metastasis.

### RNA Extraction and Quantitative Real‐Time PCR (qRT‐PCR) Assay

TRIzol reagent (CWbiotech) was used to lyse cells and extract total RNA according to the manufacturer's instructions. qRT‐PCR was performed using a 2× SYBR Green qPCR master mix (CWbiotech) and primers. The detailed primer sequences used in the study are listed in Table S2 (Supporting Information).

### Western Blotting

Cells or tissues were lysed with RIPA extraction reagent (FDBio), and the proteins were denatured at 98 °C for 20 min. Protein was subsequently separated in 8–12% SDS‐PAGE and transferred to a PVDF membrane. After that, the membrane was incubated with the primary antibodies for 12–16 h at 4 °C, followed by incubation with corresponding secondary antibodies (Jackson ImmunoResearch). The detailed information on primary antibodies used in this research is exhibited in Table S3 (Supporting Information).

### Cell Counting kit‐8 (CCK‐8) Assay

RCC cells were seeded into a 96‐well plate. To measure the cell viability, CCK‐8 (Dojindo Laboratories) was added into each well and incubated for 2 h at 37 °C under 5% CO2. The absorbance was read at 450 nm by a Multiskan FC Microplate Photometer (Thermo Scientific).

### Colony Formation Assay

RCC cells were plated in a 6‐well plate at a density of 1000 cells/well. After 9 days, colonies were fixed with 4% paraformaldehyde, stained with 0.3% crystal violet, and photographed by SYSTEM GelDoc XR (Bio‐rad).

### EdU Assay

EdU assay was performed using EdU Cell Proliferation Kit with Alexa Fluor 555 (Meilunbio). Briefly, cells were incubated with 10 µM EdU overnight. After that, the cells were fixed with 4% paraformaldehyde, incubated with 0.1% Triton‐X100 and stained with DAPI. The images were taken with a fluorescence microscope (Olympus).

### Cell Cycle Assay

A cell cycle staining kit (MultiSciences) was used according to the manufacturer's instructions. The stained cells were analyzed by flow cytometry using BD FACSCalibur (BD Biosciences).

### Transwell Assay

Migration assays were conducted using transwell 8.0 µm filters (Millipore) according to the manufacturer's instructions. After 24 h, migrated cells through the membrane were fixed using 4% paraformaldehyde and stained with 0.3% crystal violet.

### Bioinformatic Analysis

The UALCAN online database (http://ualcan.path.uab.edu) was used to analyze the expression of PCIF1 and LPP3 in RCC tumors and normal tissues.^[^ ^72^ ^]^ Datasets GSE14994 and GSE46699 were obtained from the GEO portal (https://www.ncbi.nlm.nih.gov/gds/). Proteomics data of the CPTAC RCC cohort was downloaded from its portal (https://gdc.cancer.gov/about‐gdc/contributed‐genomic‐data‐cancer‐research/clinical‐proteomic‐tumor‐analysis‐consortium‐cptac) to investigate the correlation between PCIF1 and LPP3. DAVID database (https://david.ncifcrf.gov/tools.jsp) was used to conduct functional annotation of differentially expressed genes upon PCIF1 depletion in cancer cells.

### Quantification of m^6^Am Level by LC‐MS/MS Analysis

2 µg of RNA extracted from tissues was lysed by incubating with lysis buffer containing S1 nuclease, Alkaline Phosphatase, and Phosphodiesterase I at 37 °C. Subsequently, the nucleosides were extracted and analyzed using a UPLC‐ESI‐MS/MS system. m6Am/A levels were detected by MetWare (http://www.metware.cn/) based on the ABSciex QTRAP 6500 LC‐MS/MS platform.

### m6Am‐Exo‐Seq

m6Am‐Exo‐seq was conducted based on previously reported protocal.^[^ ^11^ ^]^ Briefly, mRNA was extracted from PCIF1‐KD and control OS‐RC‐2 cells, then broken into 100–300 bp RNA fragments at 70 °C for 6 min. To enrich 5’UTR mRNA fragments, mRNA was first incubated with anti‐7‐Methylguanosine antibody and washed Dyna beads Protein G (Invitrogen) overnight at 4 °C, Immunoprecipitated RNA was collected and subsequently phosphorylated with 20U T4 PNK at 37 °C for 90 min and then dephosphorylated at 30 °C for 3 h with Terminator 5′‐Phosphate‐Dependent Exonuclease (Lucigen), treated RNA fragments were collected with Zymo RNA clean and concentrator kit (Zymo Research). After that, 3U Cap‐Clip (CellScript) was added to decap the RNA fragment, and a Zymo RNA clean and concentrator kit was used to purify and collect decapped RNA fragments. Then the second round of immunoprecipitation was performed by incubating processed RNA fragments with premixed m6A antibody (Sigma–Aldrich:ABE572), Dynabeads Protein G (Invitrogen) and Dynabeads Protein A (Invitrogen) system for 1–3 h at 4 °C, washed it with low‐salt buffer and high‐salt buffer for several times and collected them with HiPure cell miRNA Kit (Magen). After removal of ribosomal RNA, Immunoprecipitated RNA was prepared for library generation and sequencing was conducted with an Illumina Novaseq platform.

### m6A‐RNA Immunoprecipitation Assay

The m6A‐RNA immunoprecipitation experiments were performed with Magna RIP RNA‐Binding Protein Immunoprecipitation Kit (Millipore) under the guidance of the manufacturer's instructions. Briefly, cells were lysed using RIP lysis buffer and incubated with the m6A antibody (Abclonal: A17924) or IgG and protein G magnetic beads, followed by wash and protein digestion with proteinase K. The RNA was then extracted and detected by qRT‐PCR.

### Polysome Profiling Analysis

2 × 10^7^ PCIF1 KD and control RCC cells were treated with 100 µg mL^−1^ cycloheximide (Sigma) for 5 min at 37 °C and washed twice with PBS containing 100 µg mL^−1^ cycloheximide before they were collected and lysed in 400 µL polysome lysis buffer (1% Triton X‐100, 0.3 m NaCl, 15 mm MgCl_2_, 15 mm Tris‐HCl (pH 7.4), 100 µg mL^−1^ cycloheximide, 1 mm DTT and 100U RNase inhibitor) on ice for 5 min. Centrifuged at 12,000 rpm at 4 °C for 10 min, the supernatant was added to the sucrose density gradient and centrifuged at 4 °C at 38,000 rpm for 120 min (Optima XPN‐100, Beckman). The samples were then fractionated by gradient fractionator (Biocomp) with OD values at 260 nm detected. Each fraction of RNA was extracted using TRIzol (Cwbiotech) and the polysome profiling was analyzed by qRT‐PCR.

### Biotin‐dC‐Puromycin Labelling Assay

3 × 10^6^ PCIF1 KD and control RCC cells were incubated with 1:1000 Biotin‐dC‐puromycin (NU‐925‐BIO‐S, Jena Bioscience) for 12 h. Cells were then collected and lysed in 1 mL lysis buffer (1% NP40, 20 mm Tris‐HCl, PH 7.4, 150 mm NaCl, 10% glycerol, protease inhibitor cocktail). After centrifuging at 15000 rpm for 30 min at 4 °C, collect the supernatant, using 10% as input. The supernatant was then incubated with 80 µL streptavidin sepharose beads (65001, Invitrogen) by rotating at 4 °C overnight. The mixture was washed 5 times with 1% NP40 buffer and subject it to western blot analysis.

### Dual Luciferase Reporter Assay

Two luciferase reporters were constructed: pGL3‐LPP3‐WT‐5'UTR and pGL3‐LPP3‐MUT‐5'UTR, containing a 50 bp sequence from the 5' UTR of LPP3 mRNA. Vector/PCIF1 wildtype/PCIF1 mutant plasmids and *Renilla* reporter were co‐transfected into 293T cells. After 48 h, the *Firefly* and *Renilla* luciferase intensity was measured by a luminometer (Turner Biosystems). The final *Firefly* luciferase values were normalized to *Renilla* luciferase values.

### Elisa Assay

Elisa assay for phosphatidic acid was performed with a human phosphatidic Elisa kit (Jingmei Biotechnology) according to the manufacturer's instructions. Briefly, the samples/standards and HRP‐conjugate reagent were added into the testing well and incubated for 60 min at 37 °C. Then the chromogen solution was added and incubated at 37 °C for 15 min. The absorbance was read at 450 nm by a Multiskan FC Microplate Photometer (Thermo Scientific). The concentration of phosphatic acid was calculated based on the standard curve. The results were normalized to the protein content.

### Mitochondria Isolation

The mitochondria in RCC cells were isolated with a mitochondria isolation kit (Beyotime, C3601). Briefly, cells were collected and resuspended in mitochondria isolation reagent and homogenized on ice with a glass homogenizer. The homogenates were centrifuged at 600 g at 4 °C for 10 min, and the supernatants were collected and centrifuged another 600 g for 10 min for more purity. Last, the supernatants were centrifuged at 11000 g for 10 min and the pellet obtained was the isolated mitochondria.

### Quantitative Mass Spectrometry of Mitochondrial Phospholipids

Mitochondria were isolated from RCC cells as mentioned above. Lipids were extracted from samples in the presence of mixed internal standards (AVANTI, 330707‐1EA) according to the MTBE method. The final lipid samples were dissolved in 200 µL 90% isopropanol/acetonitrile and centrifuged at 14000 g for 15 min and 3 µL of the sample was injected. Mass spectra were acquired by Q‐Exactive Plus in positive and negative mode, respectively. The LipidSearch database was employed for the identification of lipid species based on MS/MS math. The lipidomics results were normalized to the protein content.

### Immunofluorescence

RCC cells were seeded on glass coverslips in 24‐well plates. The cells were fixed in 4% paraformaldehyde for 5 min and permeabilized with 0.25% Triton X‐100 for 10 min. After washing three times with PBS, the cells were incubated with primary antibody overnight at 4 °C, followed by incubation with fluorescent secondary antibody (Invitrogen) at 37 °C for 1 h. DAPI was used for nuclear staining. The cells were visualized with an Olympus BX53 fluorescence microscope.

For Mitochondrial staining, cells were incubated with 100 nM Mito‐Tracker Red CMXRos (Beyotime, C‐1049B) for 20 min at 37 °C, followed by being fixed in 4% paraformaldehyde and permeabilized with 0.25% Triton X‐100. The images were photographed using the confocal microscopy Olympus fv3000 (Olympus), and a 63 X objective lens was applied. For morphology analysis, cells were classified into three categories based on their mitochondrial morphology: “Fragmented”, “Intermediate” and “Elongated”, The classification criteria were consistent with established research.^[^ ^73^ ^]^ “Elongated” is with a majority of mitochondria in a cell forming interconnected with mitochondrial length > 10 µm; “Intermediate” is with mixed short tubular mitochondria with mitochondrial length < 10 µm in a cell; “Fragmented” is with a majority of punctiform mitochondria in a cell. The proportion of these three subtypes in different groups was scored using a 10 µm scale as a reference. Three replicates of ≈50 cells per group were included in the analysis.

### Crosslinking Assay

DSS crosslinking was conducted to evaluate the DRP‐1 oligomerization. Briefly, RCC cells were washed with PBS and incubated with 1 mm DSS (Sangon Biotech) for 30 min. Then protein was extracted and analyzed by western blotting.

### Transmission Electron Microscopy (TEM)

3 × 10^6^ Caki‐1 cells were fixed in 2.5% glutaraldehyde, followed by dehydration, embedding, sectioning, and staining for TEM observation. The mitochondria were observed and photographed on a Talos L120C (Thermo Scientific) TEM.

### Adenosine Triphosphate (ATP) Content Assay

The intracellular ATP was measured with an ATP assay kit (Beyotime, S0026). Briefly, RCC cells were lysed in lysis buffer and centrifuged at 12,000 g for 5 min at 4 °C. Luciferase intensity was measured by a luminometer (Turner Biosystems). The ATP content was adjusted for protein content in each sample.

### ROS and Mitochondrial Membrane Potential (MMP) Measurement

The intracellular ROS was detected with a ROS assay kit (Beyotime, S0033S). Briefly, cells were incubated with 10 µM 2’,7’‐Dichlorodihydrofluorescein (DCFH‐DA) (Beyotime) for 20 min at 37 °C. Then the fluorescence intensity of 2’,7’‐dichlorofluorescein (DCF) was measured by flow cytometry. For MMP measurement, cells were treated with JC‐1 solution (Beyotime, C2006) for 20 min at 37 °C. Flow cytometry was used to detect the relative content of JC‐1 monomers and aggregates to reflect the membrane potential.

### Oxygen Consumption Rate (OCR) Assays

1 × 10^4^ Caki‐1 cells were seeded in a Seahorse XF 96 cell culture microplate (Agilent). The cell culture microplate was placed in a 37 °C non‐CO2 incubator for 1 h before measurement. 1 µm oligomycin, 1 µm p‐trifluoromethoxy carbonyl cyanide phenylhydrazone (FCCP), and 0.5 µm mitochondrial complex III inhibitor rotenone/antimycin A (Rote/AA) were sequentially added into the wells and the OCR of RCC cells were analyzed using a Seahorse XFe 96 extracellular flux analyzer (Agilent).

### Statistical Analysis

Results in the study were presented as the mean ± SD and were analyzed using GraphPad prism7 (GraphPad Software). The statistical difference between the two groups was measured by a two‐tailed Student's t‐test. The correlation analysis between PCIF1 and LPP3 expression was examined by Pearson's correlation test. Statistical significance was defined as ^*^ *P* value < 0.05, ^**^ *P* value < 0.01, ^***^ *P* value < 0.001.

## Conflict of Interest

The authors declare no conflict of interest.

## Author Contributions

W.L. and Z.X. contributed equally to this work. G.L., H.H., and L.X. did conceptualization. W.L., Z.X., and L.D. performed methodology. W.L., Z.X., R.W., Z.L., H.W., Y.L., X.C., Y.L., H.X., Z.Z., Y.L and X.Y. did investigation. W.L., X.M. and F.L. perforemed formal analysis. W.L. and Z.X. curated the data. W.L. and Z.X. did visualization. W.L. and L.G. wrote the original draft. G.L., H.H., L.X., L.D., H.W., Z.L. and H.X. reviewed and did editing. G.L., H.H., and L.X. supervised the work.

## Supporting information



Supporting Information

## Data Availability

The m6Am‐exo‐seq data have been deposited in the Genome Sequence Archive in National Genomics Data Center, China National Center for Bioinformation / Beijing Institute of Genomics, Chinese Academy of Sciences (GSA‐Human: HRA006958) that are available upon reasonable request.
