# Supplementary material for: m6Am Methyltransferase PCIF1 Promotes LPP3 Mediated Phosphatidic Acid Metabolism and Renal Cell Carcinoma Progression
Source: Adv Sci (Weinh). 2024 Oct 18;11(46):2404033. doi: 10.1002/advs.202404033 (PMC11633504; doi:10.1002/advs.202404033)
Supplement: Supplementary file 1 — Supporting Information [file ADVS-11-2404033-s001.docx]

Supporting Information

**m6Am methyltransferase PCIF1 promotes LPP3 mediated phosphatidic acid metabolism and renal cell carcinoma progression**

Wenqin Luo, Zhehao Xu, Fan Li, Lifeng Ding, Ruyue Wang, Yudong Lin, Xudong Mao, Xianjiong Chen, Yang Li, Zeyi Lu, Haiyun Xie, Huan Wang, Ziwei Zhu, Yi Lu, Luying Guo, Xiaojing Yu, Liqun Xia, Housheng Hansen He, Gonghui Li.

**
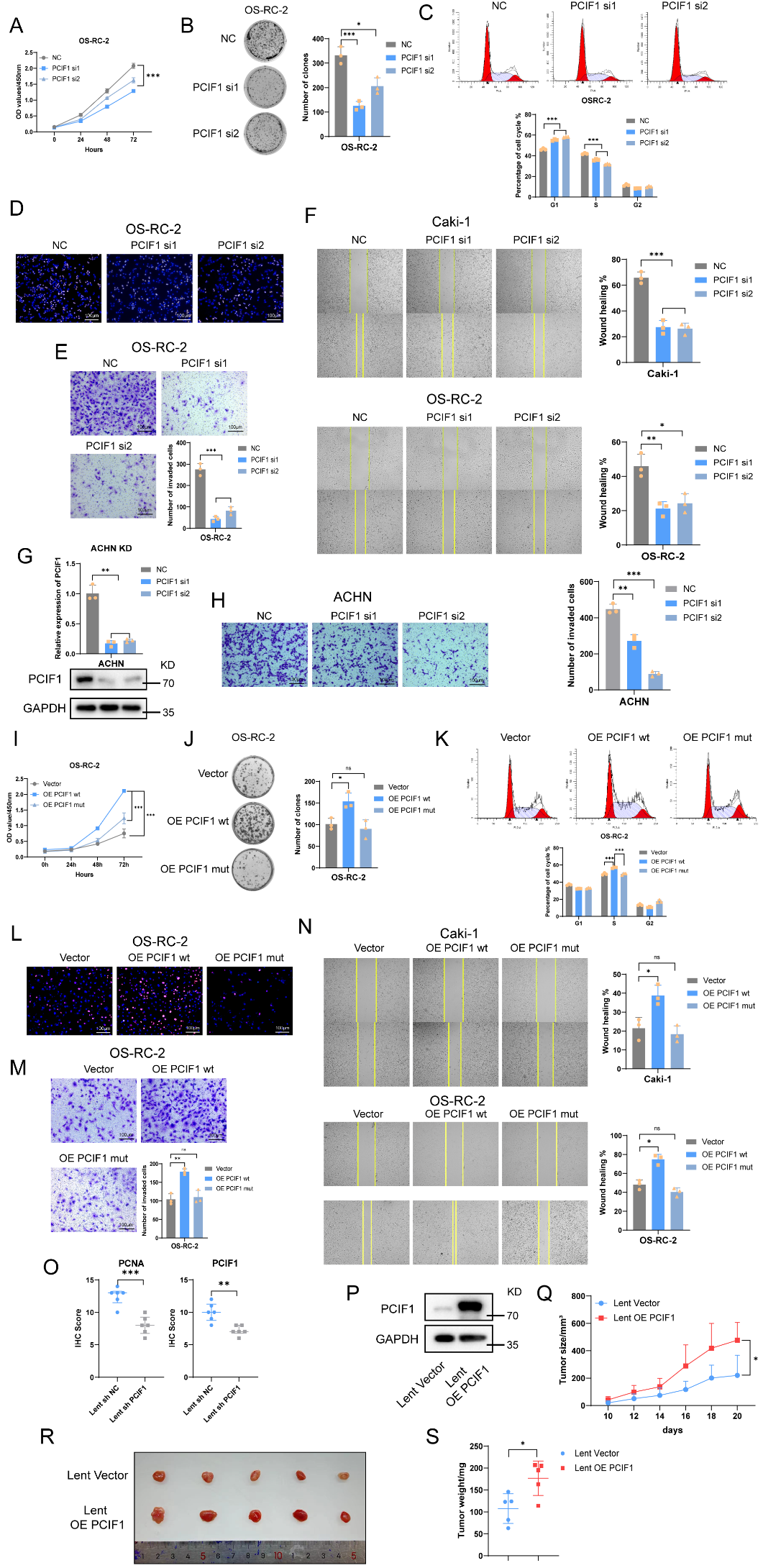
**

**Figure S1** PCIF1 is vital to RCC cells proliferation and migration

(A) Optical density at 450 nm (OD450) values of OS-RC-2 cells transfected with control or PCIF1 siRNAs in cell counting kit-8 (CCK-8) assay. (B) Representative images of clony-formation assay and its quantification data of indicated OS-RC-2 cells. (C) Flow cytometric analysis of cell cycle in OS-RC-2 cells transfected with control or PCIF1 siRNAs. (D) Representative images of EdU assay in indicated OS-RC-2 cells. Scale bar, 100 μm. (E) Representative images of transwell assay in indicated OS-RC-2 cells. Scale bar, 100 μm. The migrated cells are counted and analyzed. (F) Representative images of wound healing assay in indicated Caki-1 and OS-RC-2 cells transfected with control or PCIF1 siRNAs. (G) qRT-PCR and western blotting confirming the knockdown of PCIF1 in ACHN cells. (H) Representative images of transwell assay in indicated ACHN cells. Scale bar, 100 μm. The migrated cell is counted and analyzed. (I) Optical density at 450 nm (OD450) values of OS-RC-2 cells overexpressed with vector or wild-type/mutant PCIF1 in cell counting kit-8 (CCK-8) assay. (J) Representative images of clony-formation assay and its quantification data of indicated OS-RC-2 cells. (K) Flow cytometric analysis of cell cycle in OS-RC-2 cells transfected with vector or wild-type/mutant PCIF1. (L) Representative images of EdU assay in indicated OS-RC-2 cells. Scale bar, 100 μm. (M) Representative images of transwell assay in indicated OS-RC-2 cells. Scale bar, 100 μm. The migrated cells are counted and analyzed. (N) Representative images of wound healing assay in indicated Caki-1 and OS-RC-2 cells overexpressed with vector or wild-type/mutant PCIF1. (O) Quantification of IHC staining images for PCIF1, PCNA of OS-RC-2 cell–derived xenograft tumors. (P) Western blotting showing the PCIF1 overexpression in OS-RC-2 cells with lentivirus-based vector or PCIF1 plasmids. (Q-S) Volumes (Q), images (R) and weights (S) of indicated OS-RC-2 cell–derived xenograft tumors (n=5). *P < 0.05, **P < 0.01, ***P < 0.001; ns, not significant.


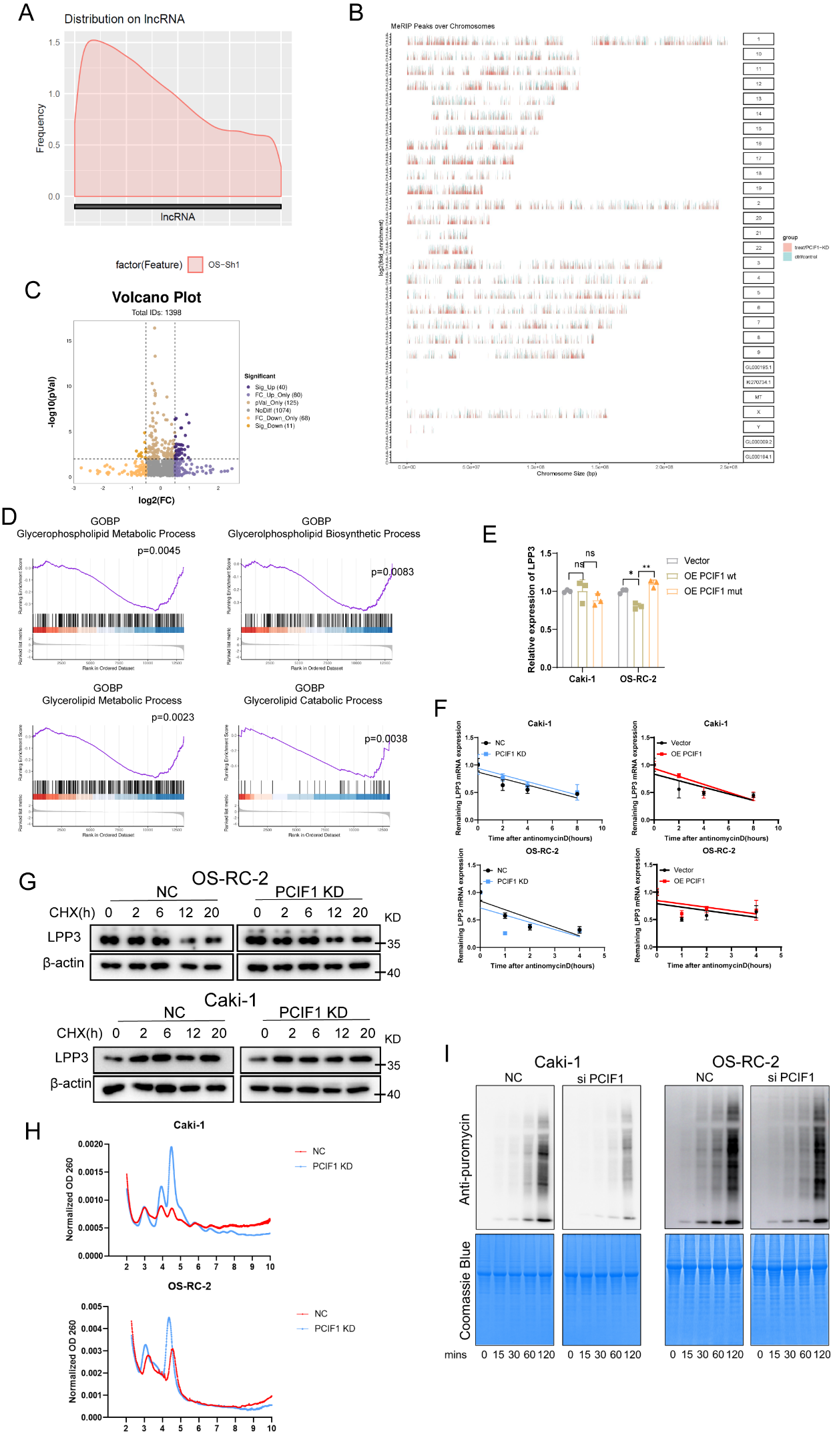


**Figure S2** PCIF1 promoted LPP3 mRNA translation via depositing of m6Am

(A) Metageneplot showing the distribution of m6Am peaks across long non-coding RNA. (B) A comprehensive map of m6Am peaks in control and PCIF1-knockdown OS-RC-2 cells across the genome. (C) Volcano plots showing the genes with differentially exhibited 5′‐UTR m6Am peaks upon PCIF1 knockdown in OS-RC-2 cells. p value <0.01, Log2FC < -0.5. (D) Gene set enrichment analysis of ‘Glycerophospholipid Metabolic Process’, ‘Glycerophospholipid Biosynthetic Process’, ‘Glycerolipid Metabolic Process’, ‘Glycerolipid Catabolic Process’ pathways upon PCIF1 knockdown. (E) qRT-PCR assay showing the mRNA level of LPP3 in RCC cells transfected with indicated plasmids. (F) qRT-PCR assay showing the remaining LPP3 mRNA levels at the indicated times in RCC cells with the silencing or overexpression of PCIF1 after actinomycin D treatment. (G) Western blotting showing the remaining LPP3 protein levels at the indicated times in control and PCIF1-knockdown RCC cells after cycloheximide treatment. (H) Polysome profiling of control and PCIF1-knockdown Caki-1 and OS-RC-2 cells. *P < 0.05, **P < 0.01, ***P < 0.001; ns, not significant. (I) Puromycin incorporation assay showing the translation efficiency of RCC cells upon PCIF1-knockdown. *P < 0.05, **P < 0.01, ***P < 0.001; ns, not significant.


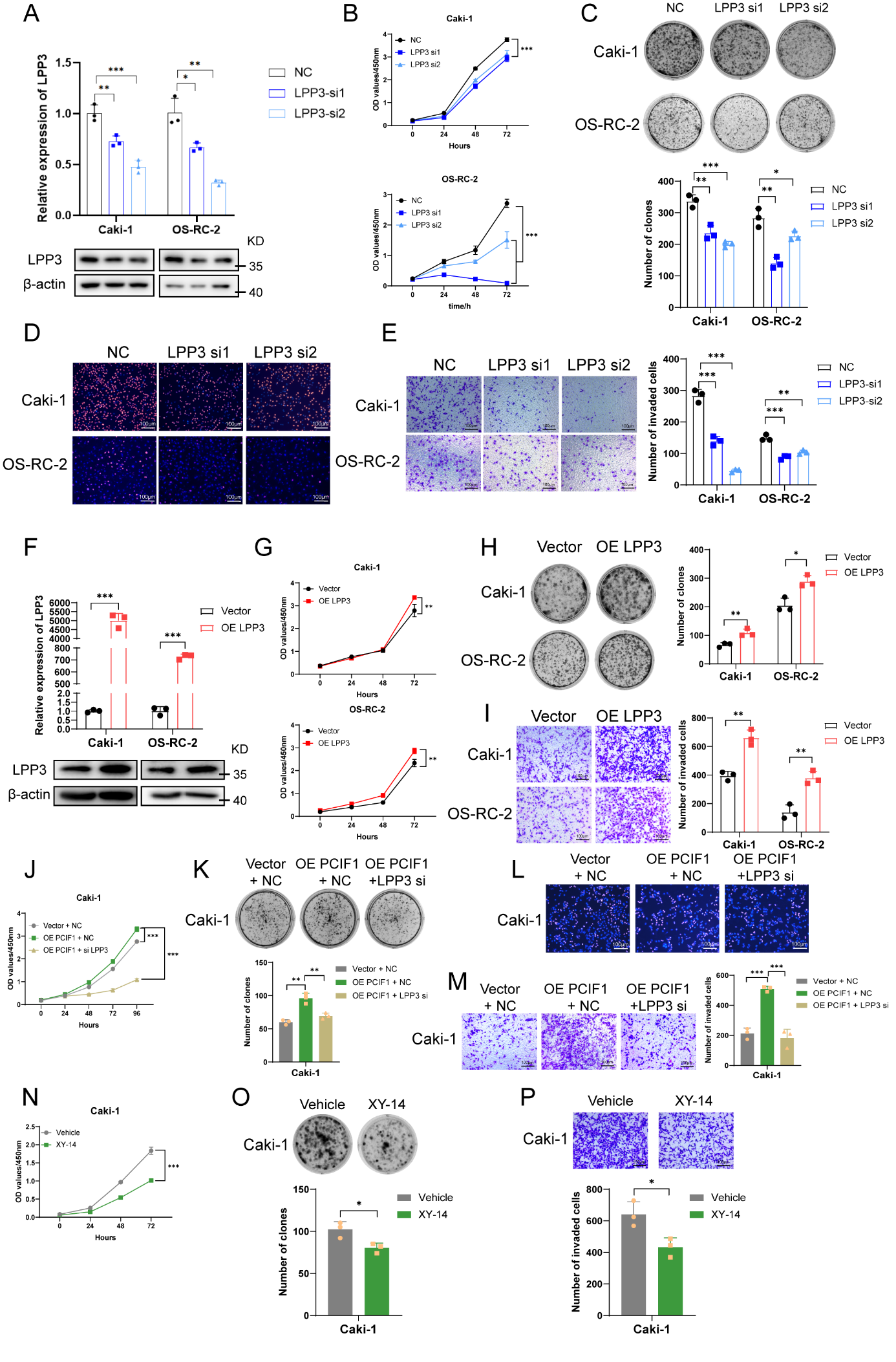


**Figure S3** LPP3 facilitated RCC cancer cells proliferation and migration

(A) qRT-PCR and western blotting confirming the knockdown of LPP3 in Caki-1 and OS-RC-2 cells. (B) Optical density at 450 nm (OD450) values of Caki-1 and OS-RC-2 cells transfected with control or LPP3 siRNAs in cell counting kit-8 (CCK-8) assay. (C) Representative images of clony-formation assay and its quantification data of indicated Caki-1 and OS-RC-2 cells. (D) Representative images of EdU assay in indicated Caki-1 and OS-RC-2 cells. Scale bar, 100 μm. (E) Representative images of transwell assay in indicated Caki-1 and OS-RC-2 cells. Scale bar, 100 μm. The migrated cells are counted and analyzed. (F) qRT-PCR and western blotting showing the overexpression of LPP3 in Caki-1 and OS-RC-2 cells. (G) Optical density at 450 nm (OD450) values of Caki-1 and OS-RC-2 cells transfected with vector or LPP3 overexpression plasmid in cell counting kit-8 (CCK-8) assay. (H) Representative images of clony-formation assay and its quantification data of indicated Caki-1 and OS-RC-2 cells. (I) Representative images of transwell assay in indicated Caki-1 and OS-RC-2 cells. Scale bar, 100 μm. The migrated cells are counted and analyzed. (J) Optical density at 450 nm (OD450) values of Caki-1 cells transfected with indicated siRNAs and plasmids in cell counting kit-8 (CCK-8) assay. (K) Representative images of clony-formation assay and its quantification data of indicated Caki-1 cells. (L) Representative images of EdU assay in Caki-1 cells transfected with indicated siRNAs and plasmids. Scale bar, 100 μm. (M) Representative images of transwell assay in indicated Caki-1 cells. Scale bar, 100 μm. The migrated cells are counted and analyzed. (N) Optical density at 450 nm (OD450) values of Caki-1 cells treated with vehicle or XY-14 (10 µM) in cell counting kit-8 (CCK-8) assay. (O) Representative images of clony-formation assay and its quantification data of indicated Caki-1 cells. (P) Representative images of transwell assay in Caki-1 cells treated with vehicle or XY-14 (10 µM). Scale bar, 100 μm. The migrated cells are counted and analyzed. *P < 0.05, **P < 0.01, ***P < 0.001; ns, not significant.


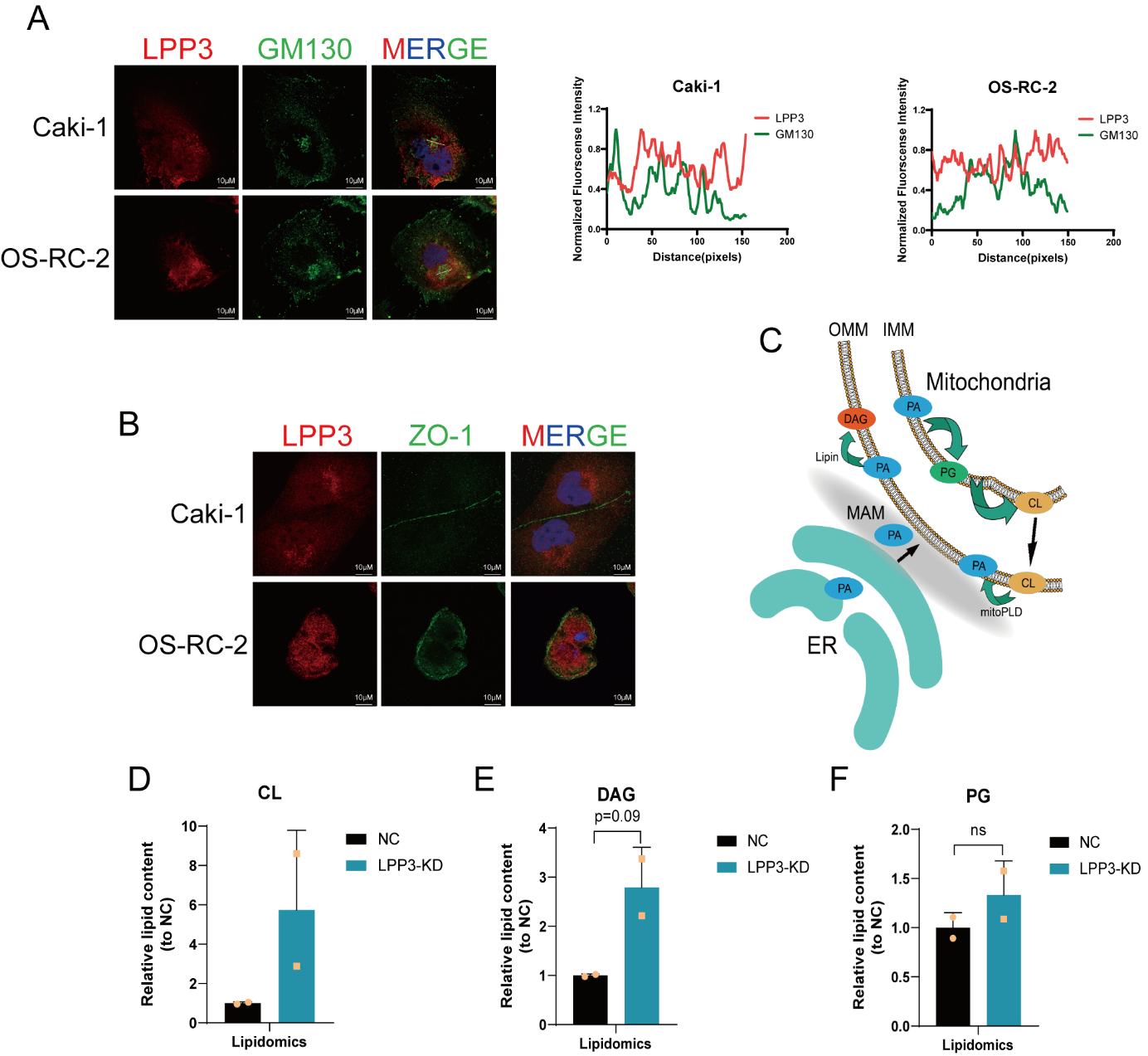


**Figure S4** LPP3 is predominantly located on ER and influences phospholipid metabolism in RCC

(A) Representative immunofluorescence images in Caki-1 and OS-RC-2 cells stained with anti-LPP3 and anti-GM130. (B) Representative immunofluorescence images in Caki-1 and OS-RC-2 cells stained with anti-LPP3 and anti-ZO-1. (C) Schematic diagram exhibiting the phospholipid metabolism pathways relating to PA in mitochondria. (D-F) Quantification of CL (D), DAG (E) and PG (F) levels in mitochondria of control and PCIF1-knockdown Caki-1 cells, relative to control condition. Data are representative of two independent experiments and are shown as the mean ± SD. *P < 0.05, **P < 0.01, ***P < 0.001; ns, not significant.

**
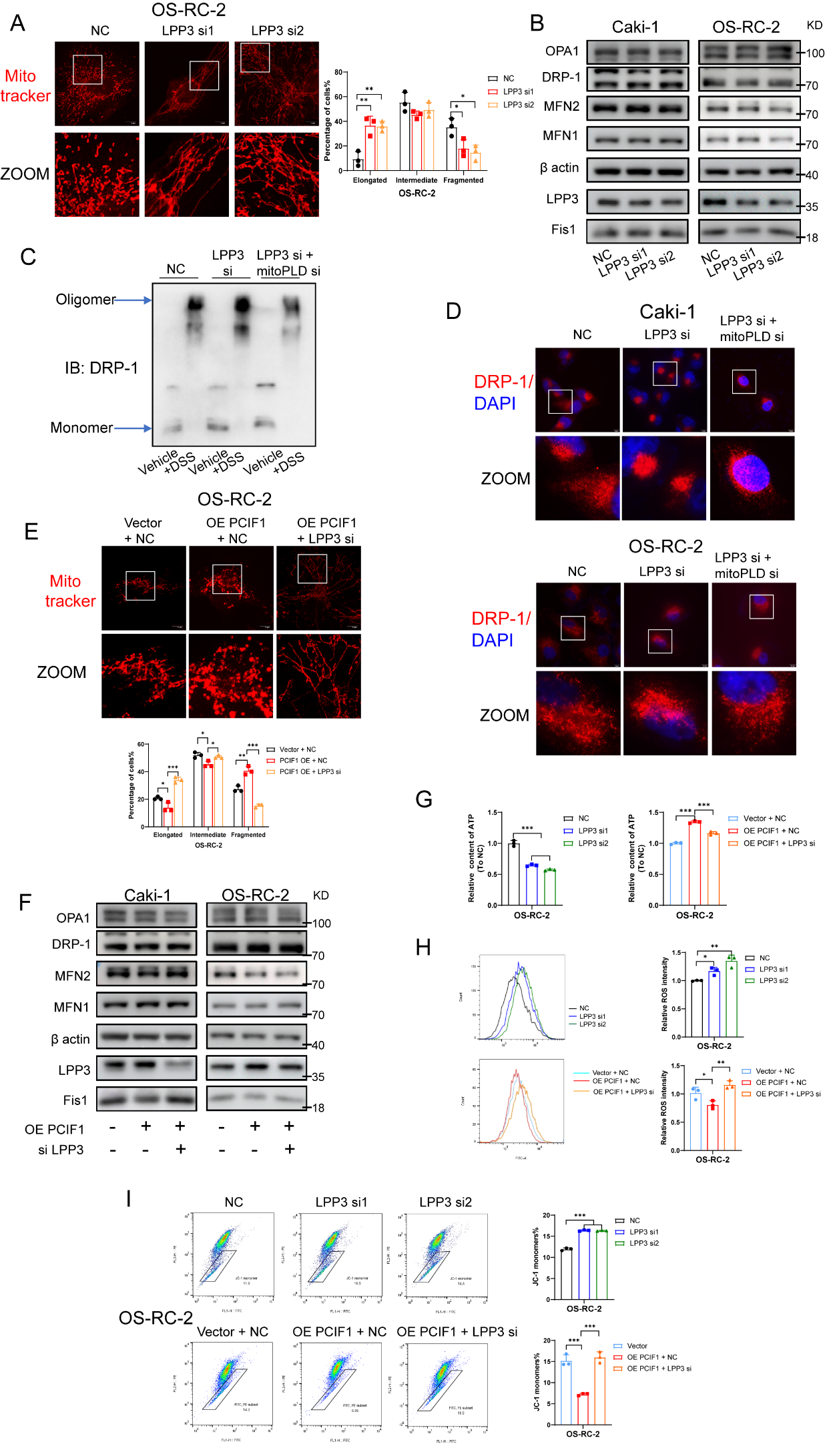
**

**Figure S5** PCIF1/LPP3 axis modulated morphology and function of mitochondria via promoting its fission

(A) Representative mitochondrial morphology stained by mitotracker in indicated OS-RC-2 cells and quantification of mitochondrial network. Scale bar, 10 μm. Around 50 cells per group were assessed. (B) Western blotting showing the expression of mitochondrial dynamics regulatory proteins in Caki-1 and OS-RC-2 cells transfected with indicated LPP3 siRNAs. (C) Western blotting showing the monomers and oligomers of DRP-1 in OS-RC-2 cells transfected with indicated siRNAs. (D) Representative immunofluorescence figures showing the DRP-1 foci in Caki-1 and OS-RC-2 cells transfected with indicated siRNAs. (E) Representative mitochondrial morphology stained by mitotracker in indicated OS-RC-2 cells with quantification of mitochondrial network. Scale bar, 10 μm. Around 50 cells per group were assessed. (F) Western blotting showing the expression of mitochondrial dynamics regulatory proteins in Caki-1 and OS-RC-2 cells transfected with indicated siRNAs and plasmids. (G) Cellular ATP levels in indicated OS-RC-2 cells. (H) Flow cytometric analysis showing ROS levels in indicated OS-RC-2 cells. The average ROS levels are calculated. (I) Flow cytometric analysis showing the mitochondrial membrane potential in indicated OS-RC-2 cells. The proportions of cells with JC-1 monomers were quantified. *P < 0.05, **P < 0.01, ***P < 0.001; ns, not significant.

**
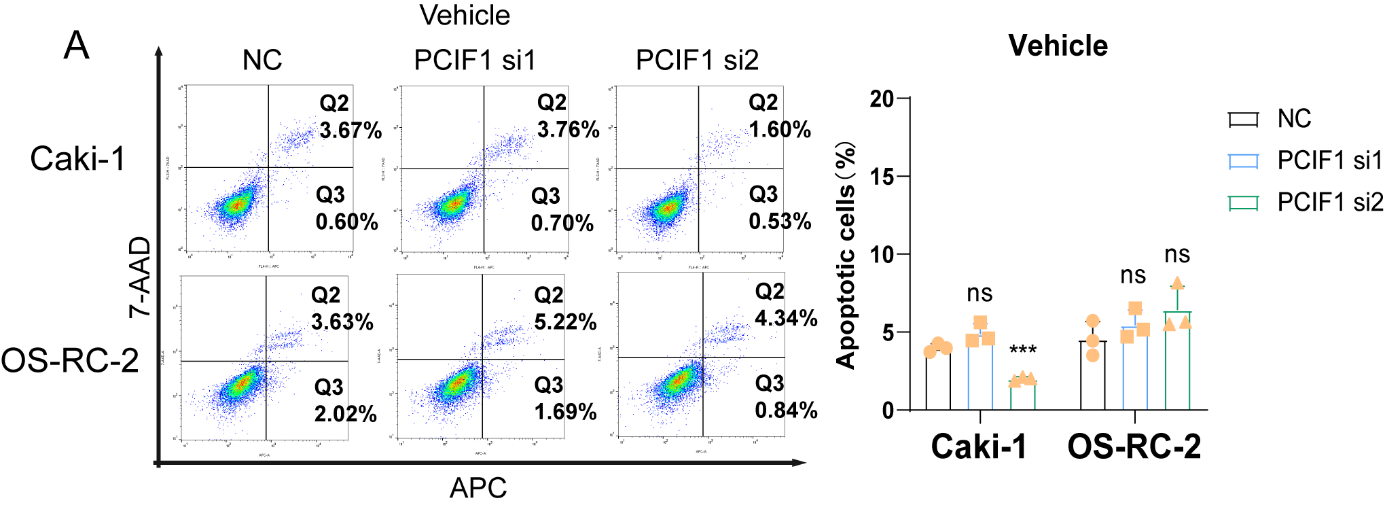
**

**Figure S6** Silencing of PCIF1 alone does not lead to an increase of cell apoptosis

(A) Flow cytometric analysis showing apoptotic ratio of Caki-1 and OS-RC-2 cells transfected with control or PCIF1 siRNAs. *P < 0.05, **P < 0.01, ***P < 0.001; ns, not significant. Data are representative of three independent experiments and are shown as the mean ± SD.

Table S1 Patients clinical informations

| NO | Gender | Age | Tumor Size | ISUP grade | Metastasis | Node |
| --- | --- | --- | --- | --- | --- | --- |
| 1 | M | 60 | 7.3*6.5 | 2 | 1 | 0 |
| 2 | M | 80 | 3.7*3.7 | 3 | 0 | 0 |
| 3 | M | 45 | 3*2.5 | 2 | 0 | 0 |
| 4 | M | 55 | 2.6*2.1 | 3 | 0 | 0 |
| 5 | M | 68 | 2*1.7 | 2 | 0 | 0 |
| 6 | M | 51 | 4*3.8 | 2 | 0 | 0 |
| 7 | M | 37 | 4.5*4.2 | 1 | 0 | 0 |
| 8 | M | 64 | 5*4.5 | 2 | 0 | 0 |
| 9 | M | 65 | 3.5*2.5 | 2 | 0 | 0 |
| 10 | M | 68 | 4.2*4 | 2 | 1 | 0 |
| 11 | F | 57 | 3.5*3 | 2 | 0 | 0 |
| 12 | F | 75 | 3.2*3 | 2 | 0 | 0 |
| 13 | M | 73 | 7.6*7 | 3 | 1 | 0 |
| 14 | M | 60 | 4*3.5 | 3 | 0 | 0 |
| 15 | M | 85 | 2.3*1.8 | 3 | 0 | 0 |
| 16 | M | 53 | 20*18 | 3 | 1 | 0 |
| 17 | M | 59 | 2*2 | 3 | 0 | 0 |
| 18 | M | 81 | 3.8*3.2 | 3 | 0 | 0 |
| 19 | F | 43 | 1.5*1.5 | 2 | 0 | 0 |
| 20 | M | 74 | 5.3*4 | 3 | 0 | 0 |
| 21 | M | 68 | 7*6 | 2 | 0 | 0 |
| 22 | F | 70 | 5*4.5 | 2 | 0 | 0 |
| 23 | M | 50 | 4*4 | 1 | 0 | 0 |
| 24 | M | 67 | 4*2.8 | 2 | 0 | 0 |
| 25 | F | 45 | 1.5*1.5 | 3 | 0 | 0 |
| 26 | M | 52 | 2.5*2 | 2 | 0 | 0 |
| 27 | M | 89 | 5.5*5 | 2 | 0 | 0 |
| 28 | M | 61 | 2*2 | 3 | 0 | 0 |
| 29 | M | 55 | 10*8 | 2 | 0 | 0 |
| 30 | M | 58 | 2.5*2.2 | 3 | 0 | 0 |
| 31 | M | 58 | 9.5*9.7 | 2 | 1 | 0 |
| 32 | F | 61 | 4*3.5 | 2 | 0 | 0 |
| 33 | M | 52 | 6*6 | 3 | 1 | 0 |
| 34 | M | 59 | 2.5*2.5 | 2 | 0 | 0 |
| 35 | F | 68 | 6*5 | 2 | 0 | 1 |
| 36 | F | 44 | 6.5*5.5 | 4 | 0 | 0 |
| 37 | M | 74 | 4.5*3 | 3 | 1 | 0 |
| 38 | F | 53 | 2*2 | 2 | 0 | 0 |
| 39 | M | 75 | 5*3 | 2 | 0 | 0 |
| 40 | M | 57 | 4*3.5 | 3 | 1 | 0 |
| 41 | M | 69 | 4.5*3.5 | 3 | 0 | 0 |
| 42 | F | 68 | 1.8*1.6 | 2 | 0 | 0 |
| 43 | F | 56 | 2.3*2.3 | 2 | 0 | 0 |
| 44 | F | 39 | 5*3.5 | 2 | 0 | 0 |
| 45 | F | 59 | 1.9*1.5 | 2 | 0 | 0 |
| 46 | M | 55 | 5.5*5 | 3 | 1 | 0 |
| 47 | M | 69 | 3.5*3 | 3 | 1 | 0 |
| 48 | M | 72 | 3.8*3.5 | 3 | 0 | 0 |
| 49 | M | 80 | 3.8*3.5 | 2 | 0 | 0 |
| 50 | M | 46 | 3.8*3.5 | 2 | 0 | 0 |
| 51 | F | 61 | 2.5*2 | 2 | 0 | 0 |
| 52 | M | 49 | 4*3.7 | 2 | 0 | 0 |
| 53 | F | 74 | 7.5*6.5 | 3 | 0 | 1 |
| 54 | M | 64 | 3*2.8 | 2 | 0 | 0 |
| 55 | M | 45 | 4.8*4.5 | 3 | 0 | 0 |
| 56 | M | 55 | 6.5*5.5 | 3 | 0 | 0 |
| 57 | F | 89 | 4.3*4 | 2 | 0 | 0 |
| 58 | F | 75 | 7.5*6.5 | 3 | 0 | 0 |
| 59 | F | 55 | 7*5 | 2 | 0 | 0 |
| 60 | F | 57 | 4.5*4 | 2 | 0 | 0 |
| 61 | M | 63 | 6.5*6 | 3 | 0 | 0 |
| 62 | F | 58 | 4.5*4.5 | 2 | 0 | 0 |
| 63 | M | 59 | 8*5 | 1 | 0 | 0 |
| 64 | M | 73 | 4.5*3.8 | 2 | 0 | 0 |
| 65 | M | 69 | 5.5*5 | 2 | 0 | 0 |
| 66 | M | 51 | 6*4 | 2 | 0 | 0 |
| 67 | M | 67 | 5*4 | 2 | 1 | 0 |
| 68 | M | 57 | 5*5 | 2 | 0 | 0 |
| 69 | F | 72 | 2*1.8 | 2 | 0 | 0 |
| 70 | M | 66 | 6*4 | 2 | 1 | 0 |
| 71 | M | 57 | 4*3.2 | 3 | 0 | 0 |
| 72 | M | 73 | 7.5*4 | 3 | 0 | 0 |
| 73 | M | 51 | 4.5*3.7 | 2 | 0 | 0 |
| 74 | M | 75 | 6.5*5.5 | 3 | 1 | 0 |
| 75 | F | 63 | 4*4 | 2 | 0 | 0 |
| 76 | F | 39 | 2.7*2 | 2 | 0 | 0 |

Table S2 Oligonucleotides

| Gene Symbol | Forward primer (5’→3’) | Reverse primer (5’→3’) |
| --- | --- | --- |
| *PCIF1* | AATCGTCCCTACTACTTCAACCG | GGTCCGAAATCACATCGTGC |
| *GAPDH* | GGAGTCAACGGATTTGGT | GTGATGGGATTTCCATTGAT |
| *LPP3* | GCGTATCAGACCACAAGCACCA | AGGGAGAGCGTCGTCTTAGTCT |

| siRNA | Anti-sense (5’→3’) |
| --- | --- |
| *NC* | AATCGTCCCTACTACTTCAACCG |
| *si PCIF1-1* | AUCUUGGGCUUCUUCACACTT |
| *si PCIF1-2* | AUGUGGUAGAUCUUACUGCTT |
| *si PCIF1-3’UTR* | UUUACAAAUGAAACAAGGUTT |
| *si LPP3-1* | UGAAAGGAUUUCCUUCCGGTT |
| *si LPP3-2* | ACUUUGCUGUCAUCACCUCTT |
| *si mito-PLD* | AUGAAAGCAAUCUCCCUCCTT |

| Rabbit anti-PCIF1 for Western and IHC | Abclonal | Cat# A18347 RRID:AB_2862117 |
| --- | --- | --- |
| Mouse anti-GAPDH for Western | Cell Signaling Technology | Cat# 2118, RRID:AB_561053 |
| Mouse anti-β-actin for Western | Proteintech | Cat# 66009-1-Ig, RRID: AB_2687938 |
| Rabbit anti-LPP3 for Western, IHC | Abclonal | Cat# A15743, RRID: AB_2763159 |
| Rabbit anti-LPP3 for IF | Abcam | Cat# AB221918 |
| Rabbit anti-m6A for RIP | Abclonal | Cat# A17924, RRID: AB_2770239 |
| Mouse anti-GRP78 for Western and IF | Proteintech | Cat# 66574-1-Ig, RRID: AB_2881934 |
| Mouse anti-GM130 for IF | Proteintech | Cat# 66662-1-Ig, RRID: AB_2882017 |
| Mouse anti-ZO-1 for IF | Proteintech | Cat# 66452-1-Ig, RRID: AB_2881821 |
| Rabbit anti-TOM20 for Western | Proteintech | Cat# 11802-1-AP, RRID: AB_2207530 |
| Rabbit anti-DRP1 for Western and IF | Abcam | Cat# ab184247 |
| Invitrogen Alexa Fluor 594 goat anti-rabbit | Invitrogen | Cat# A11011 |
| Invitrogen Alexa Fluor 488 goat anti-rabbit | Invitrogen | Cat# A11034 |
| Invitrogen Alexa Fluor 594 goat anti-mouse | Invitrogen | Cat# A11005 |
| Peroxidase AffiniPure Goat anti-mouse IgG(H+L) | Immunoreagents Inc. | Cat# 115-035-003 |
| Peroxidase AffiniPure Goat anti-rabbit IgG(H+L) | Immunoreagents Inc. | Cat# 111-035-003 |

Table S3 Antibodies
